# Characterization of Phosphorylated Peptides by Electron-Activated and Ultraviolet Dissociation Mass Spectrometry: A Comparative Study with Collision-Induced Dissociation

**DOI:** 10.1021/jasms.4c00048

**Published:** 2024-04-16

**Authors:** Marion Girod, Delphine Arquier, Amanda Helms, Kyle Juetten, Jennifer S. Brodbelt, Jérôme Lemoine, Luke MacAleese

**Affiliations:** Universite Claude Bernard Lyon 1, CNRS, Institut des Sciences Analytiques, UMR 5280, F-69100, Villeurbanne, France; Universite Claude Bernard Lyon 1, CNRS, Institut des Sciences Analytiques, UMR 5280, F-69100 Villeurbanne, France; Department of Chemistry, The University of Texas at Austin, Austin, Texas 78712, United States; Department of Chemistry, The University of Texas at Austin, Austin, Texas 78712, United States; Department of Chemistry, The University of Texas at Austin, Austin, Texas 78712, United States; Universite Claude Bernard Lyon 1, CNRS, Institut des Sciences Analytiques, UMR 5280, F-69100 Villeurbanne, France; Universite Claude Bernard Lyon 1, CNRS, Institut Lumierè Matierè, UMR5306, F-69100 Villeurbanne, France

**Keywords:** electron-induced dissociation, photofragmentation, phosphorylation modifications, fragmentation method, EAD, ETD, UVPD

## Abstract

Mass-spectrometry-based methods have made significant progress in the characterization of post-translational modifications (PTMs) in peptides and proteins; however, room remains to improve fragmentation methods. Ideal MS/MS methods are expected to simultaneously provide extensive sequence information and localization of PTM sites and retain labile PTM groups. This collection of criteria is difficult to meet, and the various activation methods available today offer different capabilities. In order to examine the specific case of phosphorylation on peptides, we investigate electron transfer dissociation (ETD), electron-activated dissociation (EAD), and 193 nm ultraviolet photodissociation (UVPD) and compare all three methods with classical collision-induced dissociation (CID). EAD and UVPD show extensive backbone fragmentation, comparable in scope to that of CID. These methods provide diverse backbone fragmentation, producing *a*/*x*, *b*/*y*, and *c*/*z* ions with substantial sequence coverages. EAD displays a high retention efficiency of the phosphate modification, attributed to its electron-mediated fragmentation mechanisms, as observed in ETD. UVPD offers reasonable retention efficiency, also allowing localization of the PTM site. EAD experiments were also performed in an LC-MS/MS workflow by analyzing phosphopeptides spiked in human plasma, and spectra allow accurate identification of the modified sites and discrimination of isomers. Based on the overall performance, EAD and 193 nm UVPD offer alternative options to CID and ETD for phosphoproteomics.

## Graphical Abstract

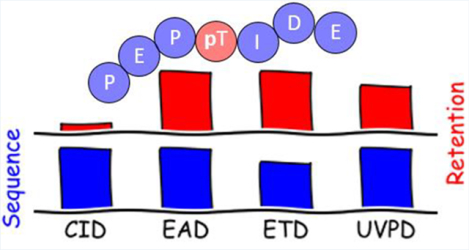

## INTRODUCTION

In the intricate world of cellular signaling, the addition and removal of phosphate groups on proteins, known as protein phosphorylation, play a pivotal role in orchestrating a wide array of biological processes.^1,2^ These reversible post-translational modifications (PTMs) affect specific amino acid residues, typically serine (S), threonine (T), and tyrosine (Y), act as molecular switches, turning on or off specific signaling pathways, and, thus, regulate the function and activity of proteins within cells. Indeed, protein phosphorylation is central to a wide range of cellular processes, including signal transduction, gene expression, cycle regulation, cell growth, differentiation, and response to external stimuli.^3,4^ Many diseases such as cancer, inflammation, metabolic disorders, and neurodegenerative diseases are also linked to kinase protein phosphorylation.^5^ Characterizing protein phosphorylation is crucial for understanding how cells respond to environmental cues, communicate with one another, and maintain homeostasis. Identification and mapping of phosphorylation on proteins present a fascinating challenge for scientists seeking to unravel the molecular intricacies in proteomics because of their low abundance, lability, and unique chemical properties.

Tandem mass spectrometry (MS/MS) has emerged as an indispensable tool, enabling the precise and comprehensive characterization of protein phosphorylation events with high accuracy, relative speed, and sensitivity.^2^ Collision-induced dissociation (CID) is widely used for the identification of phosphopeptides based on the presence of a specific phosphate loss (−80 Da HPO_3_ or −98 Da H_3_PO_4_) from the precursor ion. However, the increased neutral loss yield observed in CID hinders exact phosphorylation site localization, especially in widespread Ser/Thr-rich sequences. Metastable atom-activated dissociation (MAD) and higher-energy collision dissociation (HCD) experiments on phosphorylated peptides in negative ion mode have been used to produce more structural evidence on modified sites.^6^ Alternatively, using dual spray ion/ion reactions, CID showed significant improvement in terms of fragmentation and retention of phosphate group.^7^

Electron-driven methods such as electron capture dissociation (ECD) and electron transfer dissociation (ETD) have been developed as an alternative to CID.^8,9^ After “electron-mediated” activation, precursor ions typically yield *c* and *z* ions without side-chain loss, enabling the efficient localization of peptide phosphorylation sites.^10^ ECD and ETD methods require multiply charged ions, since the electron attachment or transfer results in charge state reduction and, thus, neutralization of singly charged analytes. Moreover, the higher the precursor charge state, generally the higher the fragmentation efficiency.^11^ However, multiply charged species can be difficult to form in the case of phosphorylated peptides due to the acidity of the phosphate groups.^12^ The use of dinuclear zinc complexes with phosphate groups, promoting multiple charge states, facilitates phosphopeptide sequencing by ETD-MS/MS.^13^ Amine-reactive TEMPO-based free radical initiated peptide sequencing (FRIPS) has been reported for negative ion mode analysis of phosphorylated peptides, generating sequence informative ions with no significant PTM loss.^14^ Moreover, as electron-driven methods are dependent on the peptide charge state, doubly protonated peptides generally suffer from lower fragmentation efficiency when compared to CID.^15^ To circumvent this issue, introduction of additional vibrational energy to the precursor ions (via collisional activation) can increase the fragmentation efficiency and sequence coverage in electron transfer with higher energy collisional dissociation (EThcD) or electron transfer with collisional activation dissociation (ETcaD),^16,17^ while also increasing the neutral phosphate losses. Phosphopeptides have also been analyzed using ETD and ECD after ESI in negative polarity, due to the acidic nature of the phosphate group, which has shown comprehensive fragmentation while retaining the PTM groups.^18,19^

As an alternative, photon-based ultraviolet photodissociation (UVPD) methods have been developed at various wavelengths for phosphoproteome characterization.^20,21^ UVPD tends to provide large arrays of peptide backbone cleavages while preserving labile post-translational modifications due to the rapid deposition of energy to the analyte. UVPD at 193 nm in negative ionization mode offers high sequence coverage and efficient H_3_PO_4_ group retention ratios during fragmentation of phosphopeptide anions.^20,22^ UVPD at 220 nm on protonated tyrosine-containing phosphopeptides showed characteristic aromatic side chain losses from tyrosine residues.^23^ Recently, spatiotemporal changes in site-specific Ser5 phosphorylation of RNA polymerase II carboxy-terminal domain have been evaluated using UVPD for sequence identification, phosphosite localization, and differentiation of phosphopeptide isomers.^24^ Infrared multiphoton dissociation (IRMPD) has been shown to be effective for differentiation of phosphorylated peptides from unphosphorylated ones,^25^ owing to the vibrational modes of PO_4_^3−^ which significantly enhance the photoabsorption cross sections of phosphorylated peptides. However, IRMPD presents the same drawbacks as thermal CID with substantial PTM losses and, as a result, ambiguous localization of phosphorylation sites. Combined UV and IR photodissociation, known as HiLoPD, is a method that provides diverse peptide backbone fragmentation with high sequence coverage while offering reasonable retention efficiency for phosphopeptides,^26^ thus enabling PTM site localization. Infrared photoactivation at 10.6 *μ*m of peptides during the ETD reaction, called AI-ETD,^16^ results in the generation of more *b*/*y* ions, which leads to the more comprehensive identification of phosphopeptides compared to ETD alone.

Recently, electron-activated dissociation (EAD) has been developed for analysis of peptides with PTMs.^27–29^ This activation method uses low-energy electrons (~1–10 eV), comparable to ECD, but is implemented on quadrupole time-of-flight (Q-TOF) systems rather than an FTICR system for which ECD was originally developed. In the present work, we evaluate the respective performances of CID, ETD, EAD, and 193 nm UVPD for complex phosphorylated peptide cation characterization. The various method performances are examined under three specific angles: (i) obtaining adequate backbone fragmentation with high sequence coverage, (ii) identifying the exact position of phosphate groups, and (iii) comparing the retention ratio of these labile groups in the fragment ions. The performance of EAD was also evaluated for phosphorylation analysis in a liquid chromatography (LC)-MS/MS workflow.

## MATERIALS AND METHODS

### Peptides and Solvents.

The synthetic phosphopeptides with 85% purity were obtained from GeneCust Europe. Sequences are MGLAFES(HPO_3_)TK, MGLAFEST(HPO_3_)K (proteotypic of the human Apolipoprotein B), DPT(HPO_3_)NGY(HPO_3_)YK (proteotypic of the human Kin of IRRE-like protein 2), TCMY(HPO_3_)GGITK (proteotypic of enterotoxin type C2 from *Staphylococcus aureus*), and ISENIS(HPO_3_)ECLYGGTTLNSEK and ISENISECLYGGT(HPO_3_)TLNSEK (proteotypic of enterotoxin type H from *Staphylococcus aureus*). All peptides were used without any further purification. Human serum was obtained from Etablissement Français du Sang. dl-Dithiothreitol (DTT), iodoacetamide (IAM), ammonium bicarbonate (AMBIC), and porcine pancreatic trypsin were purchased from Sigma-Aldrich. Optima LC-MS grade water (H_2_O), methanol (MeOH), and acetonitrile (ACN) were obtained from Fisher Chemical, and LC-MS grade formic acid (FA) was obtained from Fluka.

### Sample Preparation.

First, all peptides were prepared individually at a 200 nM concentration in H_2_O/ACN 90/10 (v/v) + 0.1% FA and directly infused at 3 *μ*L/min for mass spectrometry analysis.

100 ng of each peptide in water was spiked in 10 *μ*L of human plasma (500 *μ*g of proteins). The complex mixture was then denatured and reduced with 20 *μ*L of urea 8 M, 5.5 *μ*L of DTT 15 mM at 60 °C for 40 min, and next alkylated with 35 mM IAM at room temperature in the dark for 40 min. In order to reduce the urea concentration, samples were diluted 2-fold with AMBIC before overnight digestion at 37 °C with trypsin using a 1/30 (w/w) enzyme to substrate ratio. Digestion was stopped by the addition of formic acid at a final concentration of 0.5%. After digestion, samples were desalted and concentrated by SPE (Oasis HLB 3 cm^3^) and eluted with 1.5 mL MeOH. The eluted samples were dried at 40 °C in a N_2_ stream and resuspended in 200 *μ*L of a H_2_O/ACN (95/5, v/v) + 0.1% FA solution.

### Instrumentation and Mass Spectrometry Operating Conditions.

#### Electron-Activated Dissociation and Collision-Induced Dissociation.

MS analyses were performed on a Sciex ZenoTOF 7600 system (Darmstadt, Germany) equipped with an OptiFlow TurboV ion source and operated in positive ion mode with a Zeno trap activated. The following ion source parameters were as follows: a spray voltage of 5.5 kV, a capillary temperature of 300 °C, ion source gases 1 and 2 both set to 70 psi, curtain gas of 30 psi, and CAD gas (nitrogen) of 7 psi. The declustering potential (DP) was set at 60 V. The quadrupole resolution was adjusted to 1 ± 0.1 amu, while the resolution of the TOF analyzer was 30000.

The LC separation was carried out on a nanoEase C18 column (300 *μ*m × 150 mm, 1.8 *μ*m) from Waters. The LC mobile phase consisted of H_2_O containing FA 0.1% (v/v) used as eluent A, and ACN containing FA 0.1% (v/v) used as eluent B. Elution was performed at a flow rate of 5 *μ*L/min on an ACQUITY UHLC binary pump system (Waters). The elution sequence, for the digested plasma samples, included a linear gradient from 5% to 40% of eluent B for 42 min and then a plateau at 95% of eluent B for 4 min. The gradient was returned to the initial conditions and held there for 4 min. The injection volume was 2 *μ*L.

For collision-induced dissociation (CID) experiments, the collision energy (CE) ranged from 30 to 35 V according to the peptide with an MS/MS accumulation time in the Zeno trap of 100 ms.

For electron-activated dissociation (EAD) experiments in infusion mode, the MS/MS accumulation time in the Zeno trap was set to 100 ms. The kinetic energy (KE) of the electron beam and the reaction time were optimized for each of the seven phosphopeptides (see Figure S1) by plotting the branching ratio of all fragments that were used for peptide identification. The optimal electron KE, based on the number of sequence informative fragments, was found at 10 eV regardless of the peptide size and charge state. This is in line with the standard kinetic energy of 7 eV recommended by SCIEX.^30^ The effect of the reaction time is more pronounced at low KE and relatively minor at the optimal KE; thus, this parameter was set at 30 ms in view of a lower duty cycle in LC coupling. Under our experimental conditions the very low KE (2 eV) used in ref 29 did not yield satisfactory sequence coverages. The electron beam current was 3000 nA. The CE was reduced to 12 V in order to prevent any CID contaminating event while maintaining the ion transmission in the collision cell (below 12 V, the total ion current is drastically reduced under our instrument conditions).

The accumulation time in the Zeno trap was set at 110 ms for the parallel reaction monitoring mode (PRM) analysis when coupled with LC separation. The PRM list of precursors is available in Table S1 in the Supporting Information. The kinetic energy of the electron beam was also 10 eV and the reaction time 30 ms.

#### Electron Transfer Dissociation.

ETD experiments were performed on a Velos mass spectrometer (Thermo Fisher Scientific, San Jose, CA, USA) equipped with an electrospray ionization source and an ETD module. Ionization was achieved using electrospray in positive ionization mode with an ion spray voltage of 3.5 kV and sample infusion flow rate of 3 *μ*L/min. The sheath gas and the auxiliary gas (nitrogen) flow rates were respectively set at 35 and 10 (arbitrary unit). The ion transfer capillary temperature was 280 °C. For ETD, the radical anion selected was fluoranthene^•−^ (AGC target 6e5) and the activation time was set to 200 ms after optimization in the 80–300 ms range.

MS^3^ experiments were performed by CID activation of the charged reduced singly radical [M + 2H]^•+^ formed by ETD (200 ms) of the doubly protonated [M + 2H]^2+^ with a normalized collision energy (NCE) from 15 to 25.

#### Ultraviolet Photodissociation.

UVPD experiments were performed on an Orbitrap Fusion Lumos Tribrid mass spectrometer (Thermo Fisher Scientific) modified for UVPD in the high-pressure trap of the dual linear ion trap by the addition of an excimer laser (193 nm, 500 Hz) from Coherent, Inc. as described previously.^31^ All UVPD experiments were conducted at 120000 resolution at *m*/*z* 400, with an AGC target of 1E5, and 100 scans were averaged for each spectrum. UVPD energy varied from 2 to 3 mJ per pulse, and the number of pulses ranged from 3 to 5 pulses (corresponding to a 6–10 ms activation period).

### Data Analysis.

For CID and EAD data, the data analysis was performed using the SciexOS biotool kit for fragment ion assignments. ETD and UVPD spectra were processed using ProSight Lite software^32^ after deconvolution and deisotoping of raw files to the neutral monoisotopic masses using the Xtract algorithm provided by Thermo Scientific Inc. CID and EAD data were also analyzed with ProSight Lite after deisotoping of the peak lists exported from SciexOS. For all activation modes, 50 scans were averaged during data processing. All spectra were manually validated based on the theoretical fragment mass lists provided by Protein Prospector V6.4.9.^33^ All major ion types (a, a+1, a+2, b−1, b, b+1, b+2, c−1, c, c+1, x−1, x, x +1, x+2, y, y−1, y−2, z−1, z, and z+1) were considered. To identify the loss of PTMs, the exact masses of the labile groups HPO_3_ (79.966330 Da) and H_3_PO_4_ (97.976895 Da) were subtracted from the precursor and fragment ions containing the modified amino acids. H_2_O and NH_3_ losses from the fragment ions were also considered. Sequence coverages were calculated by using ProSight Lite.

PTM retention efficiency was calculated for each peptide by the equation

$$\text{retention}\;\text{ratio}=\text{mean}\left(\frac{\sum I_{\text{retained}}}{\sum \left(I_{\text{retained}}+I_{\text{loss}}\right)}\right)$$

where *I* is the ion intensity.

PRM data were processed using Skyline, showing the 6 transitions corresponding to the 6 main specific peptide fragments.

## RESULTS

**Figure 1.**
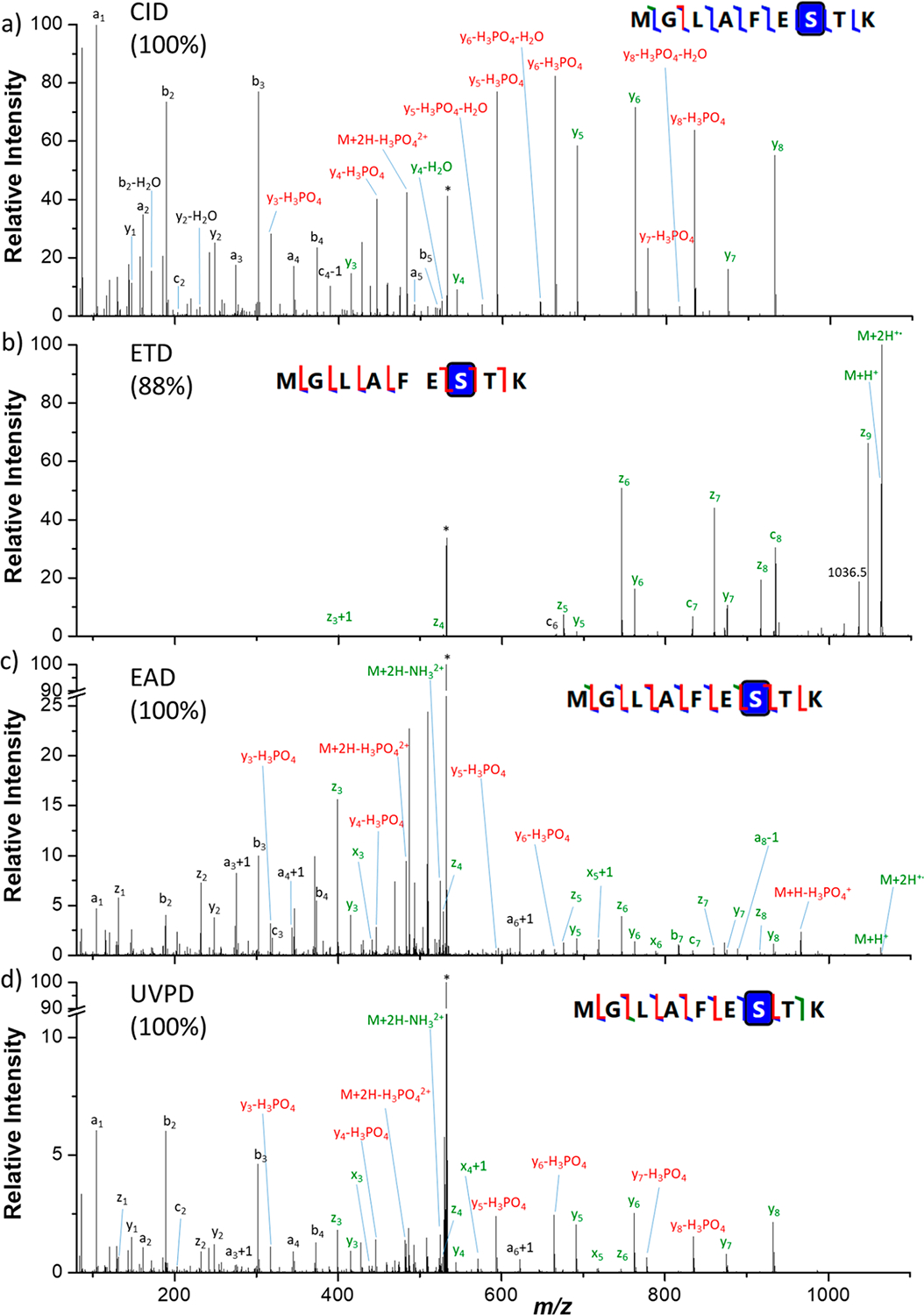
(a) CID, (b) ETD, (c) EAD, and (d) UVPD spectra of the doubly protonated [M + 2H]^2+^ (*m*/*z* 532.2301) MK9pS peptide. Precursor ions are indicated by an asterisk (*). Sequence coverages (%) are presented in parentheses. Fragments annotated in green contain intact phosphate moieties, while fragments in red have lost the modification. Detailed assignments of the fragment ions are summarized in Table S2.

**Figure 2.**
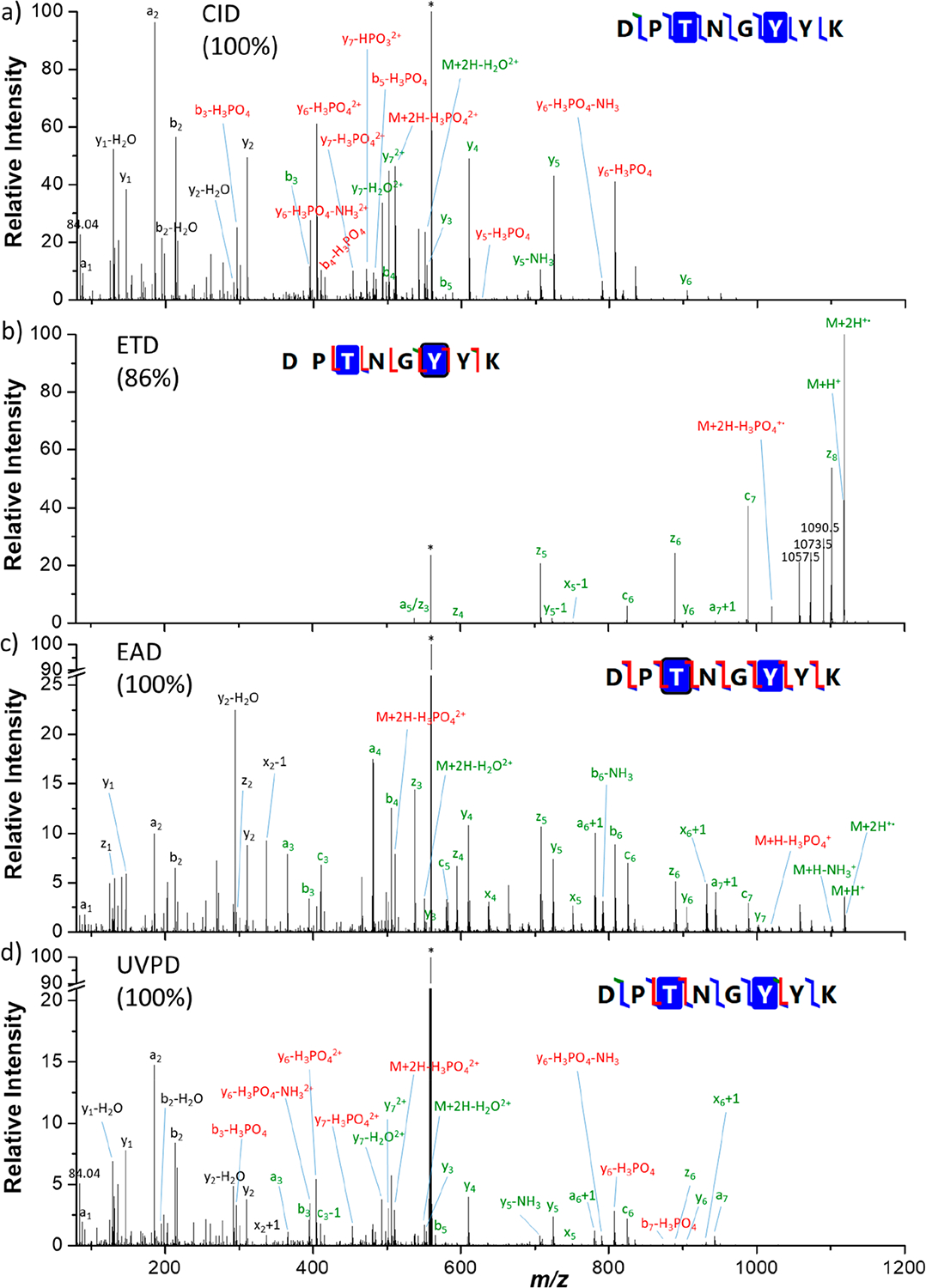
(a) CID, (b) ETD, (c) EAD, and (d) UVPD spectra of the doubly protonated [M + 2H]^2+^ (*m*/*z* 559.1872) DK8pTpY peptide. Precursor ions are indicated by an asterisk (*). Sequence coverages (%) are presented in parentheses. Fragments annotated in green contain the intact phosphate moieties, while fragments in red have lost the modification. Detailed assignments of the fragment ions are summarized in Table S3.

**Figure 3.**
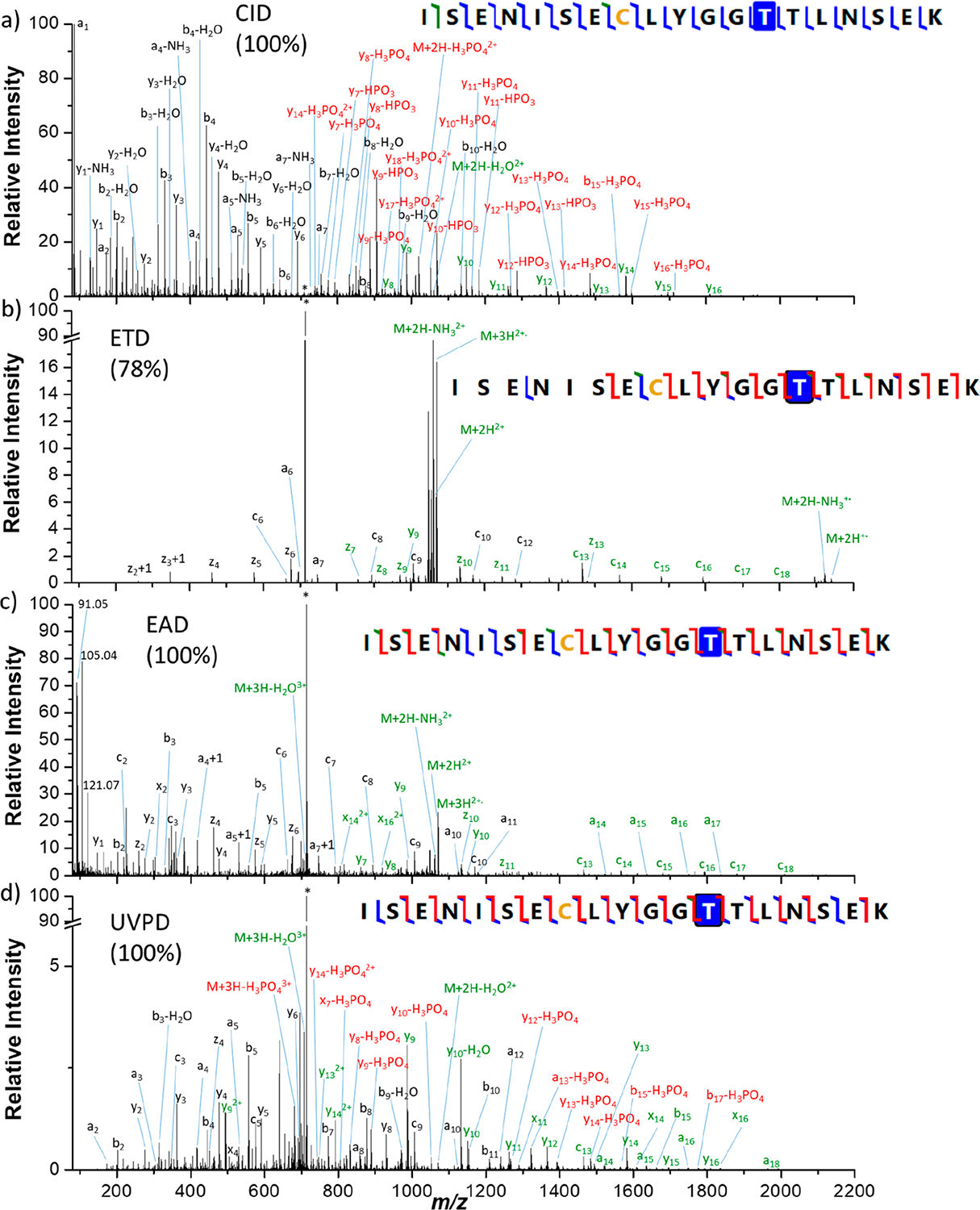
(a) CID, (b) ETD, (c) EAD, and (d) UVPD spectra of the triply protonated [M + 3H]^3+^ (*m*/*z* 713.3180) IK19pT peptide. Precursor ions are indicated by an asterisk (*). Sequence coverages (%) are presented in parentheses. Fragments annotated in green contain the intact phosphate moieties, while fragments in red have lost the modification. Detailed assignments of the fragment ions are summarized in Table S5.

First of all, MS/MS analyses were performed for six synthetic phosphopeptides. A subset of four peptides was chosen to compare after direct infusion the results obtained with different activation methods: CID, ETD, EAD, and UVPD. Spectra were recorded for the doubly protonated [M + 2H]^2+^ peptides MGLAFES(HPO_3_)TK (MK9pS), DPT(HPO_3_)NGY(HPO_3_)YK (DK8pTpY), ISENIS(HPO_3_)ECLYGGTTLNSEK (IK19pS), and ISENISECLYGGT(HPO_3_)TLNSEK (IK19pT) as well as for the triply charged peptides IK19pS and IK19pT. For all peptides, the presented spectra correspond to the most abundant precursor charge state. All spectra are described in detail in the Supporting Information and theoretical *m*/*z*, observed *m*/*z*, and assignments for all fragment ions are listed in Tables S1–S4 (errors are below 10 ppm). Figures 1–3 show spectra for peptides MK9pS^2+^, DK8pTpY^2+^, and IK19pT^3+^ with a brief description of their major features. Fragment ions that contain the initially phosphorylated site and are detected with the intact phosphate group are indicated with green labels, while those detected after H_3_PO_4_ or HPO_3_ loss bear red labels.

Then, all phosphopeptides were spiked in a complex human plasma matrix at a concentration mimicking the endogenous level to implement the phosphorylation analysis by EAD in an LC-MS/MS workflow.

### Activation of Phosphorylated MGLAFES(HPO_3_)TK Peptide (MK9pS).

The CID, ETD, EAD, and UVPD spectra of the doubly protonated [M + 2H]^2+^ (*m*/*z* 532.2301) peptide MK9pS are presented in Figure 1. In CID (Figure 1a), backbone fragmentation produces mainly *y* ions (19 fragments), some *b* ions (5) and a ions (5), and a few c ions (2). The doubly charged precursor with a neutral loss of 97.9769 Da is also observed at *m*/*z* 483.2403, which corresponds to the elimination of the H_3_PO_4_ group. The neutral loss of H_2_O is detected from most *y*_*n*_ fragments as well as b_2_. All *y*_*n*_ ions that include the initially phosphorylated S (pS) in position 7 are detected both with intact phosphate group and after H_3_PO_4_ elimination or with combined phosphate and water losses (Table S2). The CID spectrum includes sequence information in the low *m*/*z* region and provides a sequence coverage of 100%.

In ETD, the major fragment ion corresponds to the charged reduced radical ion [M + 2H]^•+^ at *m*/*z* 1064.52 and some singly charged [M + H]^+^ are also observed at *m*/*z* 1063.56. (Figure 1b). Note that we cannot exclude a contribution of the ^13^C peak of [M + H]^+^, but the relative intensity of this peak being twice higher than the intensity of [M + H]^+^ confirms the assignment of the charge reduced radical ion. Fragmentation of the peptide backbone yields fragment ions *z* (6), *y* (5) and *c* (3) ions (Table S2). The phosphate group is always preserved. Low *m*/*z* ions are not detected. The ETD sequence coverage is 88%.

EAD yields all fragment types: *a*, *b*, *c*, *x*, *y*, and *z* (Figure 1c and Table S2). Among these, the major fragments are *z* (8) and *y* (7), followed by *a* (5) and *b* (4) ions, but a few *x* (3) and *c* (2) ions are also observed. Less traditional fragment types are also detected: a+1/x+1/z+1 and a–1 ions. Singly charged [M + H]^+^ and charge reduced radical [M + 2H]^•+^ ions are observed, respectively at *m*/*z* 1063.4580 and *m*/*z* 1064.4660. Assignment of the radical [M + 2H]^•+^ ion rather than the ^13^C peak of the [M + H]^+^ is confirmed by exact masses and relative intensity in the isotopic pattern. Elimination of H_3_PO_4_ (97.9769 Da) is observed from the doubly charged precursor and from the [M + H]^+^ ion, but all pS-containing fragment ions are observed exclusively with intact phosphate group at the exception of few small *y*_*n*_ ions (*n* = 3–6) that are also observed with H_3_PO_4_ neutral loss. Neutral loss of NH_3_ (17.0265 Da) is also detected at *m*/*z* 523.7189 from the precursor ion. The EAD spectrum provides 100% sequence coverage with a complete series of *z* ions.

UVPD produces a total of 39 fragment ions (Figure 1d), which is similar to the number of fragment ions detected in EAD. Major ions are *y* (8) and *z* (7), followed by *a* (5) and *b* (5) ions. Some *x* (3) and *c* (2) ions are also observed. The spectrum also contains a+1 and c–1 ions. Similar to CID, phosphate losses are observed for all pS-containing *y* ions (Table S2). Moreover, the elimination of H_3_PO_4_ and ammonia from the doubly charged precursor ion are observed at *m*/*z* 483.2419 and *m*/*z* 523.7189, respectively. The UVPD spectrum affords 100% sequence coverage with a complete series of *y* ions.

### Activation of Doubly Phosphorylated DPT(HPO_3_)NGY(HPO_3_)YK Peptide (DK8pTpY).

The CID, ETD, EAD, and UVPD spectra of doubly protonated [M + 2H]^2+^ (*m*/*z* 559.1872) peptide DK8pTpY are presented in Figure 2.

In CID, a complete series of *y* ions is detected. Additionally, four *b* ions are observed together with two *a* ions (including the intense fragment a_2_). The precursor and all fragments are detected both intact and after elimination of the phosphate group, H_2_O or NH_3_. Moreover, elimination of HPO_3_ (79.966330 Da) is observed from the doubly charged y_7_ ion. Sequence coverage of 100% is reached.

In ETD, the charge reduced radical ion [M + 2H]^•+^ of *m*/*z* 1118.48 and the singly charged [M + H]^+^ *m*/*z* 1117.48 are prominent. Only high mass *z*_*n*_ (*n* = 3−8) ions are detected as well as abundant c_6_ and c_7_, while only a few low-abundance *a*/*x* and *y* ions are also produced. The phosphate group is preserved for all fragments, but phosphate loss is observed from the charge-reduced radical. The ETD spectrum enabled 86% sequence coverage.

In EAD, complete series of *y* and *z* ions are detected, together with *b* (6), *x* (5) and *c* (5) ions. a+1 and x+1 ions are also detected. The precursor and the charge-reduced [M + H]^+^ ion are detected both intact and after elimination of the phosphate group, H_2_O, or NH_3_. Phosphate groups are preserved for all backbone fragment ions containing the modification. A sequence coverage of 100% is reached.

In UVPD complete series of *y* and *a* ions are detected, together with *b* (6), *x* (3), and *c* (2) ions. a+1 and x+1 ions are also detected. The precursor and all pT-containing fragments are detected both intact and after elimination of the phosphate group, H_2_O, or NH_3_. A sequence coverage of 100% is reached.

### Activation of Large Phosphorylated ISENIS(HPO_3_)ECLYGGTTLNSEK (IK19pS) and ISENISECLYGGT(HPO_3_)TLNSEK (IK19pT) Peptides.

The CID, ETD, EAD, and UVPD spectra of the triply protonated [M + 3H]^3+^ (*m*/*z* 713.3180) peptides IK19pS and IK19pT are presented in Figure S1 and Figure 3, respectively.

In CID, numerous *b* (20/18 for pS/pT) and *y* (43/48) ions (including PTM losses) were detected for both peptides (Figure S1a and Figure 3a). A few *a* ions (6/8) are also detected. Neutral losses of H_3_PO_4_, water, or ammonia are observed from the doubly charged precursor ion and for several *a* and *b*/*y* fragment ions. Loss of HPO_3_ is also observed from many *y* fragments of the pT peptide. Most of the fragment ions are detected both with and without the phosphate group, but fragments detected exclusively without the modification are more numerous in the case of pS than for pT peptide (Tables S4 and S5). No fragment is detected exclusively with the intact modification. Sequence coverage of 100% with a substantial number of fragment ions are obtained for the two peptides.

ETD of these triply charged peptides mainly yields the doubly charged radical [M + 3H]^•2+^ as well as the deprotonated species [M + 2H]^2+^, but low-intensity singly charged radical [M + 2H]^•+^ ions are also observed. NH_3_ elimination is observed for the last two (Figure S1b and Figure 3b). Mainly *z* (14/12 for pS/pT) and *c* (10/12) ions are detected. A few *a* (4/2) and *y* (1/3) ions are also produced (Tables S4 and S5). The phosphate group is fully preserved for all fragment ions that included the initially modified site (pT or pS). A sequence coverage of 78% is obtained from the ETD spectra of both IK19pS and IK19pT peptides.

In EAD, all fragment ion types are generated (*a*, *b*, *c*, *x*, *y*, and *z*) (Figure S1c, Figure 3c, and Tables S4 and S5) for both peptides. The most abundant fragments are *y* (29/20 for pS/pT), *a* (15/17), and *c* (16/15) ions as well as *z* (11/11) ions in the low *m*/*z* range. Moreover, a+1 and x+1 fragments are also observed. The charge reduced radical species [M + 3H]^•2+^ is also observed at *m*/*z* 1069.9804 for both peptides. Additionally, neutral losses of H_2_O and NH_3_ are observed from the precursor ion, the doubly charged product ion [M + 2H]^2+^ and some *a*, *b*/*y* backbone fragments. No loss of phosphate group is observed from the precursor ion or from the fragment ions that include the initially phosphorylated site. A sequence coverage of 100% is reached.

In UVPD, all types of fragment ions are detected with mainly *y* (24/42 for pS/pT), *b* (20/26), and *a* (12/21) ions. A few x+1 values are also observed. Neutral loss of H_2_O and NH_3_ is observed from some *a* and *b*/*y* backbone fragments, as well as the precursor ion. The loss of the H_3_PO_4_ group is observed from a significant number of singly and doubly charged backbone fragment ions (13/22 for pS/pT) as well as from the precursor. Only very few fragments (2/1) are detected only after dephosphorylation. However, a significant number of *a*, *c*, and *x* but even *b* and *y* fragments (15/18) are observed exclusively with the intact modification (see Tables S4 and S5). Overall, a complete sequence coverage of 100% is obtained from the UVPD spectrum of IK19pT, and 89% is obtained for the IK19pS peptide.

### EAD Activation of a Pool of Phosphopeptides in Complex Matrix.

**Figure 4.**
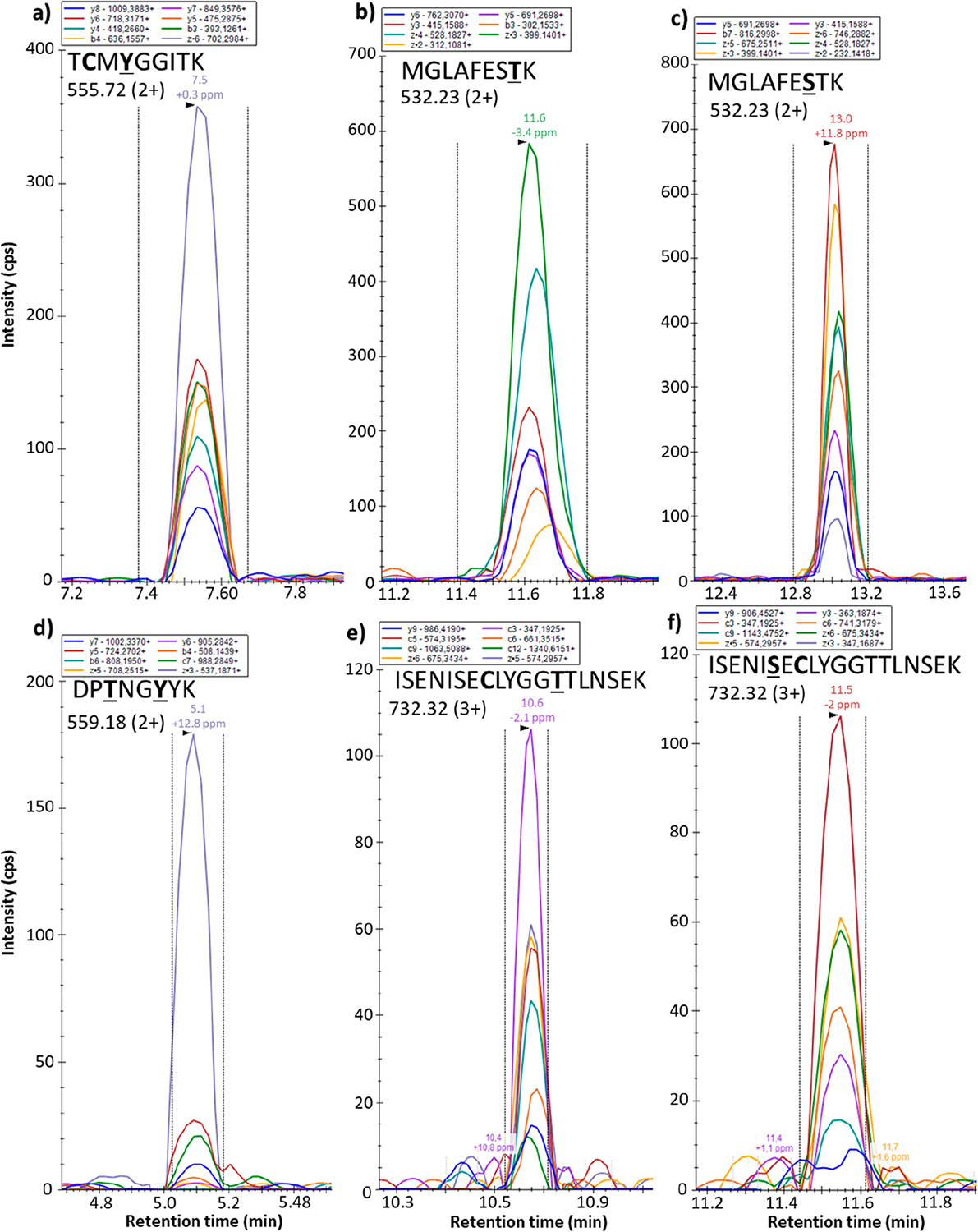
Extracted ion chromatograms of 7−8 main fragment ions detected for identified doubly protonated (a) TK9pY (*m*/*z* 555.7216), (b) MK9pT (*m*/*z* 532.2301), (c) MK9pS (*m*/*z* 532.2301), and (d) DK8pTpY (*m*/*z* 559.1872) and triply protonated (e) IK19pT (*m*/*z* 732.3240) and (f) IK19pT (*m*/*z* 732.3240) phosphopeptides spiked in the plasma matrix analyzed after carbamethylation and digestion by LC-PRM-EAD.

A pool of six synthetic phosphopeptides containing 4 isomers was spiked in a human plasma sample at a concentration of 100 ng/500 *μ*g of plasma proteins, to mimic the biological phosphorylation level.^34,35^ After digestion, the complex mixture was separated by LC and the (carbamido-methylated, when cysteine is present in the sequence) phosphopeptides were fragmented by EAD in a targeted PRM experiment (Table S1). Figure 4 shows the ion chromatograms of fragment ions extracted from the LC-PRM EAD data for the six phosphorylated peptides using Skyline. Phosphopeptides are eluted between 5 and 13 min with *z*, *b*, and *y* ions as most intense fragments. The fragment ions containing the initial PTM site can be detected only with the intact phosphate group, except for MK9pS and MK9pT peptides, which present some small degree of phosphate loss. For quantification, chromatographic peaks corresponding to fragment ions of the modified peptides were integrated and the areas of the seven most intense summed with Skyline (Table S1).

## DISCUSSION

### Type of Fragmentation and Fragmentation Efficiency.

As expected, collision activation of phosphopeptides mainly induces bond dissociations at the most labile amide (C–N) bonds after slow heating of the precursor ions and generates *b* and *y* ions. In ETD, the initial electron transfer yields the charge-reduced radical species [M + 2H]^•+^ and [M +3H]^•2+^, respectively, from doubly and triply charged precursor ions. These radical phosphopeptide product ions then break at N–C_*α*_ bonds and yield even-electron *c* ions (from the N-terminal) and odd-electron radical *z* ions (from the C-terminal). These are general features of nonergodic electron capture/transfer methods, already well described in the literature.^36^ Compared to CID and ETD, which yield preferentially one type of fragmentation channel, EAD and UVPD yield a significant number of fragment ions from all *a*/*x*, *b*/*y*, and *c*/*z* series. UVPD proceeds by transition to excited electronic states, followed by either direct (nonergodic) fragmentation or fragmentation in the ground state after internal conversion (i.e., vibrational activation).^37^ Under our experimental conditions (10 eV electrons, 50 ms), EAD is similar to hot ECD activation.^36,38^ As a result, a significant part of the activation comes from the ion–electron interaction that yields vibrationally hot and/or electronically excited ions (up to ionized precursors) prior to, in parallel, or post electron capture. The vibrational internal energy increase explains the appearance of “ergodic” *b*/*y* ions. Fewer of these “CID-type” *y* fragment ions are detected in EAD compared to UVPD. However, EAD activation also proceeds by electron capture that yields charge-reduced ions and subsequent *c/z* fragment ions, similarly to what is observed in ETD. We can note that in the case of the IK19pS and IK19pT triply charged precursors, the doubly charge-reduced radical [M + 2H]^•+^ resulting from 2 electron attachments and 1 H atom loss is observed from ETD but not observed from EAD, probably due to more internal energy deposition by EAD resulting in lower survival yield of this charge-reduced species. Alternatively, [M + 2H]^•+^ could be formed by single electron attachment to the [M + 2H]^2+^ species selectively formed after a proton transfer reaction between the precursor and the ETD reagent,^39,40^ which is not possible in EAD.

Regarding the fragmentation efficiency, under the present experimental conditions, the fragment ion signal is much lower in EAD (SciexZeno/10 eV, 30–50 ms) and UVPD (ThermoFusion/193 nm, 3 mJ, 4 pulses) than in CID (SciexZeno/30–35 V) for all peptides. This may correlate with the number of precursor ions activated (variable overlap between the electron/photon beam and the ion cloud) and the respective activation cross sections. Fragmentation yields are comparable in ETD and CID for the 8–9 amino acid peptides, but for the large peptides, ETD seems less efficient. This is probably due to the size of the peptide acting as an energy buffer and hindering, without additional heating, the disassembly of fragment ions held together by noncovalent interactions.^41^ Interestingly, the fragmentation signal is higher in EAD for the triply protonated IK19pS/pT peptides (~20–30% of the precursor ion signal, 88 fragments) compared to their doubly charged forms (<5% of the precursor ion signal with only 44 fragment ions) (see Figure S2b in the Supporting Information). This likely illustrates the higher interaction between the (negatively charged) electron beam and the positively charged peptides as their charge increases, associated with higher energy transfer and electron capture yields. Moreover, specifically for doubly charged precursors, since the charge reduced intermediate bears a single charge, only one of the complementary *c*/*z* fragments can be detected at a time. As expected, the same charge effect is observed for ETD (Figure S2a, with poor 28% sequence coverage for the 2+ precursor), in contrast to UVPD (Figure S2c), where the ion charge does not influence their interaction with photons and the photoactivation efficiency is unaffected. In terms of precursor ion size at given charge state, all activation methods show the same trend (also similar to the trend observed in CID): the larger the peptide, the smaller the fragmentation yield. The effect is more pronounced for ETD than other methods, due to the lower overall internal energy transferred to the precursor ions. In the case of the larger peptide isomers IK19pS/pT in EAD, the enhanced fragmentation by effect of charge compensates the moderate size effect (lower fragmentation efficiencies), in contrast to both ETD where the size effect is prominent, and UVPD where no charge effect is observed. As a result, the fragmentation efficiency of EAD for large/charged peptides increases by 40% (Figure S1) and becomes closer to CID (branching ratio of all fragments that were used for peptide identification are respectively 0.95/0.99 in EAD/CID spectra from Figure 3a,c). The required EAD reaction time is much shorter (30 ms) than for ETD (200 ms), which reflects the higher fragmentation efficiency resulting from a combined vibrational and electronic activation.

### Sequence Coverage.

**Figure 5.**
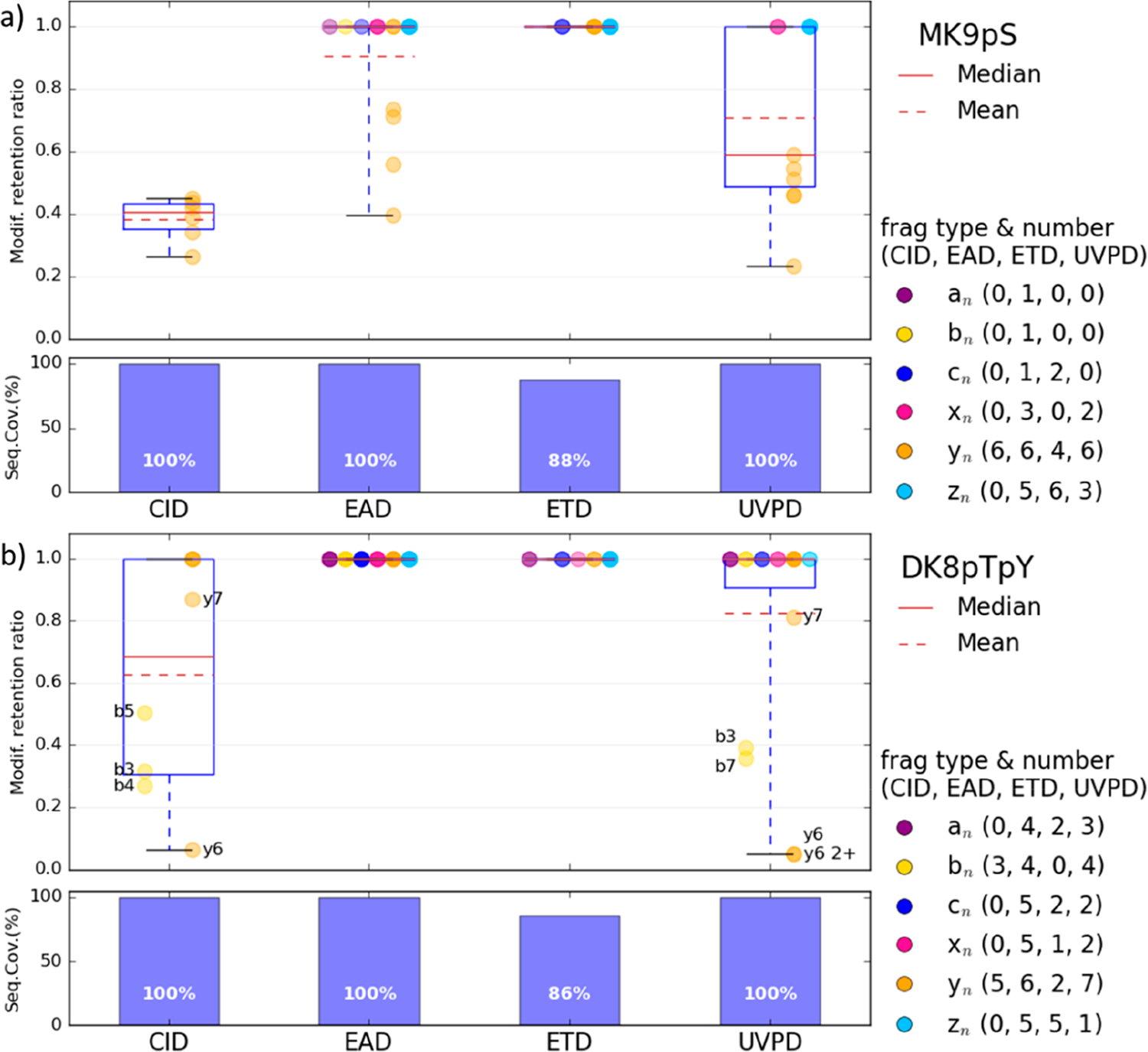
Modification retention ratios for each fragment containing the modified AA (colored by fragment type) and sequence coverages obtained in CID, ETD, EAD, and UVPD for the doubly protonated [M + 2H]^2+^ (a) MK9pT and (b) DK8pTpY peptides with labels for fragments displaying retention <0.9. The numbers in parentheses correspond to the number of each fragment type containing the modified AA for the different activation modes. The median and mean are calculated from all fragments containing the modified AA of the peptide.

**Figure 6.**
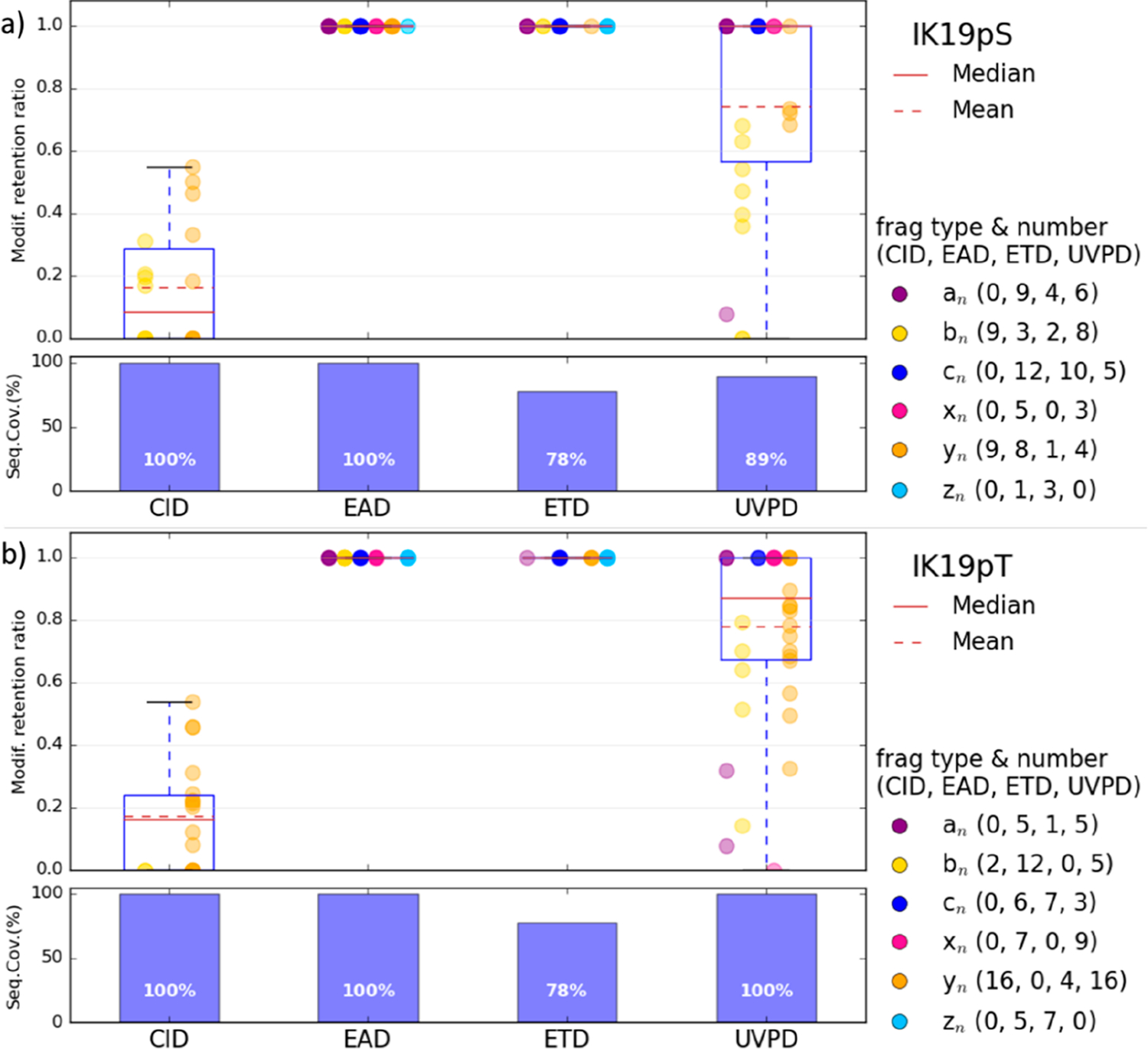
Modification retention ratios for each fragment containing the modified AA (colored by fragment type) and sequence coverages obtained in CID, ETD, EAD, and UVPD for the triply protonated [M + 3H]^3+^ (a) IK19pS and (b) IK19pT peptides. The numbers in parentheses correspond to the number of each fragment type containing the modified AA for the different activation modes. The median and mean are calculated from all fragments containing the modified AA of the peptide.

Sequence coverages of 100% are obtained in CID and EAD for all peptides (see the bottom of Figures 5 and 6). Even if a complete series of ions is not observed, the complementarity of C-terminal and N-terminal fragments allow characterization of the whole sequence. UVPD gives also high sequence coverages for the main peptides with only a slightly lower 89% for the larger IK19pS peptide. In ETD, the sequence coverage is always lower because the main process is electron attachment, and then there is not enough energy to fragment the peptide backbone (Figures 5 and 6). This becomes problematic for larger peptides (IK19pS and IK19pT) where the coverage is below 80%. To overcome this limitation, it is possible to perform MS^3^ experiments with CID on the radical species [M + 2H]^•+^ generated by ETD to give more energy for backbone fragmentation. This not only improves the sequence coverage but also results in the loss of the PTM group, as illustrated in Figure S3 for the MK9pS peptide, and reduces the compatibility of the approach with LC coupling.

### Phosphorylation Response to the Activation.

In terms of PTM identification and localization, the modification retention ratio of the activation method is particularly important. The collision activation of phosphopeptide molecular ions induces cleavage of the C–O–P ester bridge. If the cleavage of the C–O bond occurs with hydrogen transfer, phosphoric acid (H_3_PO_4_) is lost whereas breaking of the O–P bond promotes the loss of HPO_3_ (phosphite group). When the phosphite group is lost, the mass of the resulting ion (precursor ions, HPO_3_) corresponds to the nonmodified sequence. For instance, fragment y_7_-HPO_3_ of IK19pT is identical to the mass of fragment y_7_ of the nonphosphorylated IK19. The H_3_PO_4_ loss also yields fragments with mass similar to the dehydrated ions of the nonphosphorylated sequence (see y_7_-H_3_PO_4_ of IK19pT vs y_7_-H_2_O from IK19). None of these fragments allow for the localization of the initial PTM group. From the different analyzed peptides, we mainly observed H_3_PO_4_ losses and only a few HPO_3_ losses for IK19pT (*y* ions) and DK8pTpY (y_7_^2+^ ion) precursor ions.

To evaluate the ability of each activation method to retain the phosphorylation, we calculated a retention ratio for each backbone fragment ion containing the initially modified amino acid (AA) (all individual charge states and H_2_O or NH_3_ losses were considered): the intensity of the intact precursor divided by the sum of intact and −H_3_PO_4_/−HPO_3_ forms. Then for each peptide, a global mean retention ratio is calculated by averaging all of these individual retention yields (see Materials and Methods). These values are shown in Figures 5 and 6 for all peptides and all activation modes (values can be found in Tables S6 and S7). The retention ratio is plotted according to the fragment type.

As expected, in ETD, no PTM loss is ever observed from any backbone fragment (H_3_PO_4_ loss in only observed from the radical species of DK8pTpY peptide; see Table S3), making it a benchmark approach for retention of phosphorylation. The mean retention ratio is also high for EAD, where only a few side-chain losses are observed in the case of the MK9pS peptide. For all the other sequences examined, the retention ratio was maximal for EAD. In CID, the labile phosphate group elimination is favored, which yields low retention ratios. In the case of UVPD, the abundance of *b*/*y* ions tends to reduce the global retention ratio observed.

For the MK9pS peptide (Figure 5a), the mean retention percentage increases in the order 39 < 67 < 87 < 100% with CID, UVPD, EAD, and ETD, respectively.

DK8pTpY presents two modified sites on different AA; the global mean retention percentages follow the same trend with 63.2 < 81.1 < 100 < 100% in CID, UVPD, EAD, and ETD, respectively. For all activation methods, the retention ratio is greater for the phosphorylated tyrosine (pY) (99% in average) than for the phosphorylated threonine (pT) (Figure 5b). Thus, fragment ions containing only the initially modified Y site (y_3–5_) display a maximal retention ratio, while low retention ratio fragment ions (b_3–5_ and y_6,7_) are those containing the initially modified T site. This is consistent with reports in the literature where the neutral loss of phosphate groups is less systematically observed from tyrosine, compared to pS and pT, thanks to the high phosphate–tyrosine binding energy and steric hindrance on mobile proton mechanisms, both due to the aromatic group.^42^

For the large IK19pT/pS peptides, the retention ratio is 100% in EAD and ETD, while it is only 77.4%/74.3% in UVPD and even lower values in CID (16.7%/15.8%). For these peptides, typically larger backbone fragments have a higher retention yield than low *m*/*z* ones (Table S7). It seems however that the position of the PTM has little influence in this case, although it has been reported that the environment of the modified AA potentially alters the phosphate loss yield following mobile proton mechanisms in CID.^43^

The HPO_3_/H_3_PO_4_ losses are observed on b and y ions for all phosphopeptides and on some a and x ions specifically for IK19pS/pT peptides in CID and UVPD. Thus, CID, which produces essentially *b*/*y* ions, is by nature not particularly well adapted to phosphorylation (or PTM) analysis. Overall, b/y fragments are highly susceptible to be produced with low retention ratios, whichever activation method is used. However, UVPD *b*/*y* fragments do not display maximal retention but do yield significantly greater retention ratios than CID. And even more notably, EAD *b*/*y* fragments quasi-systematically display very high and up to maximal retention ratios. Apart from *b*/*y*, all other fragments display in general a very high/maximal retention ratio even with an electron kinetic energy of 10 eV. In particular, no such loss is observed from *c* and *z* ions on any peptides when produced regardless of the activation mode. The associated radical induced fragmentation mechanisms are fast and do not require equilibration of the internal energy over the whole system; thus, the labile character of the phosphate groups is no longer relevant to mediate the fragmentation.^44^ Thus, all methods producing abundant *a*/*x* and especially *c*/*z* ions are particularly interesting for PTM analysis: ETD, EAD, and UVPD allow the preservation of the labile phosphate group. Hence, EAD seems an optimal method of enabling full retention of the labile phosphate groups, whichever fragment ion type is concerned.

Moreover, especially for larger peptides IK19pS/pT, CID and UVPD spectra are more complex due to H_3_PO_4_ and HPO_3_ losses, which can confound the identification.

### Localization of the PTM Sites.

For complete characterization of the PTMs, in addition to sequence coverage, the phosphorylation site must be assigned. Although CID is routinely utilized to confirm the presence of phospho groups by detection of neutral HPO_3_/H_3_PO_4_ losses from precursor ions, the accurate localization of the modification requires the detection of sequential backbone fragment ions containing the intact modification. This is particularly true when several possible modified sites are present in the sequence, where even phosphosite localization tools (most commonly used are phosphoRS and Mascot/Andromeda) may experience significant levels of false localization rates.^15^ For a phosphopeptide containing n AA with the modification at position *m*, the unambiguous identification of the phosphorylation site requires the joint detection of N-terminal phosphorylated a_*m*/_b_*m*_/c_*m*_ ions and nonphosphorylated a_*m*–1/_b_*m*–1_/c_*m*–1_ ions (i.e., fragmentation at and just before the modified AA) or that of the C-terminal phosphorylated x_*n*–*m*–1/_y_*n*–*m*–1_/z_*n*–*m*–1_ ions and nonphosphorylated x_*n*–*m*/_y_*n*–*m*_/z_*n*–*m*_ ions (i.e., fragmentation at and just after the modified AA). Consequently, the full characterization of the PTM requires a method with both high sequence coverage and a high (maximal) retention ratio.

MK9pS peptide has two possible phosphorylated sites in positions S7 or T8; the detection of both b_6_/phospho_b_7_ and y_2_/phospho_y_3_ pairs allows the position of phosphate on S7 to be confirmed using CID, EAD, and UVPD activation modes. By ETD, no low C-terminal fragments are detected; thus, the modification was only localized by the observation of the c_6_ and phospho_c_7_ pair.

DK8pTsY peptide has three possible phosphorylated sites at position T3, Y6, or Y7. The detection of the b_2_/monophospho_b_3_ and monophospho_y_5_/diphospho_y_6_ pairs allows assignment of the position of phosphate groups on T3 and Y6, respectively, using CID, EAD, and UVPD. On the C-terminal side, y_2_/monophospho_y_3_ ions confirm the position of a phosphate group on Y6 but no further fragment confirms the second site. By ETD, no low *m*/*z* fragment ion is detected. Thus, all detected fragment ions contain at least one initially modified AA; however, the detection of diphospho_c_6_ allows the position of one phosphate on Y6 to be assigned rather than Y7.

IK19pS/pT peptides have six possible sites S2, S6, Y10, T13, T14, and S17. For IK19pS, in CID and UVPD, we observe nonmodified N-terminal fragments at I5 (b_5_, a_5_), which excludes S2, but no N-terminal S6 fragments with the intact phosphate. Indeed, the phosphate group addition is observed only for b_8_ and up to b_15_ (with H_3_PO_4_), which excludes Y10, T13, T14, and S17. As a result, even if fragments with the intact PTM are not detected at the exact phosphorylated AA, in this case, it is still possible to identify pS6 because there are no other possible phospho-sites between positions 6 and 8. From the C-terminal side, nonmodified y_1–13_ and phospho_y_14–17_ (both intact and −H_3_PO_4_ forms) are detected, confirming that the phosphate group was attached to the serine at position 6 from the N-terminal. In ETD, only phospho_a_6_ is detected (no ion without the modified AA is detected) but the detection of the y-type fragment phospho_y_14_ allow differentiating the S2 and S6 sites. In EAD, the pair y_13_/phospho_y_14_ is detected, unambiguously pinpointing the pS6 modification.

For IK19pT in UVPD, nonmodified C-terminal T6 fragments (y_6_, x_6_) are observed but no phospho_C-terminal T7 fragment. The intact phosphate group is detected only for y_8_ and after. Since AA12 (contained in phospho_ y_8_) is a G, the position of the phosphate group on T13 can be confirmed but with low confidence because nonmodified y_6_ could be total H_3_PO_4_ loss from pT14. From the N-terminal side, nonmodified N-terminal G12 fragment ions (a_12_, b_12_) and phosphorylated N-terminal T13 fragments (a_13_, c_13_) are detected, which allow support of the modification localization on T13. However, in CID for the same peptide, y_7_ is detected with and without H_3_PO_4_ modification but b_14_ is only observed without the modification, so the localization is ambiguous and could also point to pT14. In contrast, nonmodified c_12_/phospho_c_13_ are detected in ETD and EAD for IK19pT (as well as nonmodified y_6_/phospho_y_7_ in EAD only), allowing the unambiguous identification of pT13.

The localization is of course more straightforward when no phosphate loss is detected like in EAD and ETD (using the pairs on fragment ions before and after the modified AA) but is in any case limited by the overall sequence coverage. For ETD, for instance, the exact localization can be difficult for large peptides with multiple sites, because the sequence coverage is not complete. Moreover, localization of PTMs close to the end terminals can be limited due to the absence of low *m*/*z* fragment ions. Due to lower retention efficiency, the exact localization of the PTM can be more difficult in CID and UVPD modes for unknown peptides with multiple potential sites and sites close to each other.

### EAD Performance in a LC-MS/MS Workflow.

While it has been documented that UVPD analysis of PTMs is compatible with an LC-MS/MS workflow,^45^ the use of EAD is less documented. To reproduce the challenge in biology for exact localization of the phosphorylation when there are multiple possible PTM sites, a mixture of six phosphopeptides spiked in human plasma was analyzed by targeted PRM-EAD after LC separation. The concentration of the phosphorylated peptides reflects the endogenous human level, where it is estimated that one-third of all human proteins is phosphorylated at any point in time.^34,35^ The extracted ion chromatograms of the most intense *z*, *b*, and *y* fragment ions (Figure 4) show peaks with a very good signal-to-noise ratio (S/N), indicating that the EAD spectral data with 30 ms reaction time are compatible with an LC time scale.

Moreover, given the high PTM retention observed in EAD, the spectra allow for accurate identification of the modified site and discrimination of isomers. Specifically, fragment ions b_3_/y_4_ (both without modification) and b_4_/z_6_ (both containing the intact phosphate group) pinpoint the position of PTM at Y4 in TK9pY (Figure 4a). Similarly, a modified b_6_ fragment ion allows discrimination of Y7 versus Y8 for the localization of the second PTM on the DK8pTpY peptide (Figure 4d). The MK9pS/pT isomers are separated by LC: MK9pT elutes at 11.6 min while MK9pS is detected at 13 min. Indeed, b_7_ and z_2_ ions provide direct evidence for the phosphorylation site localization, as they contain or lack modification according to the isomer (Figure 4b,c). IK19pS/pT isomers, closely eluted, are also unambiguously differentiated thanks to the PTM-specific c_9_ and y_9_ fragment ions (Figure 4e,f). Overall, LC-PRM EAD data led to the successful identification of all phosphopeptides based on the detection of differentiating ions.

Additionally, the high S/N ratio of the PRM-EAD data allows accurate quantification of the phosphopeptides by integration of the chromatographic peaks.

## CONCLUSION

In this work, we report on the characterization of four phosphorylated peptides of different sizes (8 and 19 AA) and charge states (2+/3+) using mass spectrometry. Four activation methods (CID, ETD, EAD, and UVPD) were compared in terms of their ability to provide information on the peptide sequence (sequence coverage) and on the phosphorylation site (retention ratio).

The routinely used CID method is very efficient in terms of fragmentation yield and systematically provides high sequence coverage. Also, despite low retention ratios, the occurrence of intact fragments, even with low intensities, is generally sufficient to identify and localize the phosphorylation sites. Some ambiguities, however, might remain, especially with multiply phosphorylated sequences, where even phosphosite localization software might fail. ETD is frequently dedicated to PTM analysis due to its systematic maximal modification retention ratio. This well-known feature is, however, counteracted by underoptimal sequence coverage properties that may still lead to ambiguities on the phosphorylation site localization, especially for phosphorylation close to the C-/N-terms (where fewer fragments are generated) and for sequences containing multiple neighboring AA prone to phosphorylation.

In contrast to these approaches, alternative methods such as UVPD and EAD share the characteristics and considerable advantages of balancing vibrational and electronic excitations, which leads to the efficient generation of both ergodic and nonergodic fragments. Thus, UVPD produces all *a*/*x*, *b*/*y*, and *c*/*z* fragment ion types, enhancing both the sequence coverage and the global retention ratio. From this point of view, UVPD presents the joint characteristics of CID and ETD. However, the retention ratio of b/y fragments generated by UVPD is still nonmaximal but significantly higher than for CID *b*/*y* fragments. UVPD produces the richest fragmentation spectra of all methods examined (which also contributes to spectral congestion), but its retention ratios are difficult to predict due to the limited control of the amount of energy transferred.

Finally, the EAD approach, which is very similar to hot ECD, seems to be particularly interesting, as it also generates all types of fragment ions, which allows high sequence coverage and quasi-systematically preserves the labile PTM groups, even for the EAD *b*/*y* ions. These attributes allow efficient phosphosite disambiguation with regards to all other methods examined here. EAD shares many interesting properties with UVPD but offers more facile control of the amount of energy transferred by EAD, leading to slightly less rich fragmentation spectra but with maximal modification retention ratios for all types of fragments. Thus, EAD may be the method of choice for the complete characterization of phosphorylation since it enables full retention of the labile phosphate groups for all fragment types including *b*/*y* ions, and complete sequence coverage allowing the accurate determination of the position of the phosphorylation site, especially for large peptides.

The analysis of phosphopeptides in a complex human plasma sample in LC coupling configuration was performed by PRM-EAD with a sensitivity performance compatible with quantification. Moreover, on the chromatography time scale, EAD unambiguously differentiates positional isomers with accurate localization of the PTM site owing to the preservation of the phosphate groups on the fragment ions.

## Supplementary Material

Supporting
