# Supplementary material for: Characterization of Phosphorylated Peptides by Electron-Activated and Ultraviolet Dissociation Mass Spectrometry: A Comparative Study with Collision-Induced Dissociation
Source: J Am Soc Mass Spectrom. Author manuscript; Available in PMC 2025 May 1. (PMC11382297; doi:10.1021/jasms.4c00048)
Supplement: Supporting [file NIHMS2020685-supplement-Supporting.pdf]

Supporting Information

Characterization of phosphorylated peptides by electron activated and ultraviolet dissociation mass spectrometry: a comparative study with collision induced dissociation

Marion Girod*,[1] Delphine Arquier,[1] Amanda Helms,[2] Kyle Juetten,[2] Jennifer S. Brodbelt,[2] Jérôme Lemoine[1] and Luke MacAleese[3]

[1] Universite Claude Bernard Lyon 1, CNRS, Institut des Sciences Analytiques, UMR 5280, 5 rue de la Doua, F-69100 VILLEURBANNE, France

[2] Department of Chemistry, The University of Texas at Austin, Austin, TX 78712

[3] Universite Claude Bernard Lyon 1, CNRS, Institut Lumière Matière, UMR5306, F-69100, Villeurbanne, France

Correspondence to: Marion Girod, Email : marion.girod@univ-lyon1.fr

Table of Contents

Full description of Figures 1-3 and S2........S-2

Figure S1. ....S-6

Figure S2. ....S-7

Figure S3. ....S-9

Figure S4. ....S-10

Table S1. ....S-11

Table S2. ....S-12

Table S3. ....S-14

Table S4. ....S-16

Table S5. ....S-20

Table S6. ....S-25

Table S7. ....S-26

Activation of phosphorylated MGLAFES(HPO_3)TK peptide (MK9pS)

The CID, ETD, EAD and UVPD spectra of the doubly protonated $[M+2H]^{2+}$ (*m/z* 532.2301) peptide MGLAFES(H_2PO_4)TK (MK9pS) are presented in Figure 1. Theoretical *m/z*, observed *m/z*, and assignments for fragment ions detected in each experiment for this peptide are summarized in Table S1.

In CID (Figure 1a), backbone fragmentation producing mainly *y* ions (19 fragments), *b* ions (5) and *a* ions (5) is observed (Table S1). Some *c* ions (2) are also observed. The doubly charged precursor with a neutral loss of 97.9769 Da is also observed at *m/z* 483.2403, which corresponds to the elimination of the H_3PO_4 group. The neutral loss of H_2O is detected from fragment ions y_n (n=2, 3, 5, 6, 8) and b_2. Fragment ions y_n ions (n=3-8), which include the initially phosphorylated S (pS) in position 7, are detected both with intact phosphate group (in green in Figure 1a) and after H_3PO_4 elimination (in red in Figure 1a). Combined phosphate and water losses are also observed from y_5, y_6 and y_8 ions (Table S1). The CID spectrum provides sequence coverage of 100%, including sequence information in the low *m/z* region.

In ETD, the major fragment ion corresponds to the charged reduced radical ion $[M+2H]^{+\bullet}$ at *m/z* 1064.52 (Figure 1b). Some singly charged $[M+H]^+$ are also observed at *m/z* 1063.56. Fragmentation of the peptide backbone yields fragment ions *z* (6), *y* (5) and *c* (3) ions (Table S1). The phosphate group is preserved for all fragment ions containing the modification (in green in Figure 1b). The ETD spectrum provides a sequence coverage of 88%, since low *m/z* ions are not detected.

EAD of this peptide yields a wider range of fragmentation types, *a*, *b*, *c*, *x*, *y*, and *z* (Figure 1c and Table S1). Among these, the major fragments are *z* (8) and *y* (7), followed by *a* (5) and *b* (4) ions. Some *x* (3) and *c* (2) ions are also observed. All pS-containing fragment ions are observed with intact phosphate group (in green in Figure 1c). Only y_n (n=3-6) ions are also observed with H_3PO_4 neutral loss (in red in Figure 1c). Beside the traditional *a*/*x*, *b*/*y* and *c*/*z* ions, other fragment types are also detected: a+1/x+1/z+1 and a-1 ions. Singly charged $[M+H]^+$ ion is observed at *m/z* 1063.4580 as well as low charge reduced radical ion $[M+2H]^{+\bullet}$ at *m/z* 1064.4660. The elimination of H_3PO_4 (97.9769 Da) is observed from the doubly charged precursor and from the $[M+H]^+$ ion giving rise to the ions at *m/z* 483.2403 and *m/z* 965.4812, respectively. The neutral loss of NH_3 (17.0265 Da) from the precursor ion is also detected at *m/z* 523.7189. The EAD spectrum provides 100% sequence coverage with complete series of *z* ions.

UVPD with 4 laser pulses on the same peptide produces a total of 41 fragment ions (Figure 1d), which is similar to the number of fragment ions detected in EAD. Major ions are *y* (8) and *z* (7),

followed by *a* (5) and *b* (5) ions. Some *x* (3) and *c* (2) ions are also observed. In addition to the traditional ion types, the spectrum also contains a+1 and c-1 ions as well as water neutral loss from the b_2 ion. Similar to CID, phosphate losses are observed for all pS-containing y_n (n=3-8) ions (Table S1). Moreover, the elimination of H_3PO_4 and ammonia from the doubly charged precursor ion are observed at *m/z* 483.2419 and *m/z* 523.7189, respectively. The UVPD spectrum affords 100% sequence coverage with complete series of *y* ions.

Activation of doubly phosphorylated DPT(HPO_3)NGY(HPO_3)YK peptide (DK8pTpY)

The CID, ETD, EAD and UVPD spectra of the doubly protonated $[M+2H]^{2+}$ (*m/z* 559.1872) peptide DPT(H_2PO_4)NGY(H_2PO_4)YK (DK8pTpY) are presented in Figure 2. Theoretical *m/z*, observed *m/z*, and assignments for fragment ions detected in each experiment for this peptide are summarized in Table S2.

The CID, EAD and UVPD spectra provided sequence coverage of 100% with complete series of *y* (and *z* in case of EAD or *a* in UVPD) ions. The same number of *b* (6) ions (excluding phosphate losses) is observed in EAD (Figure 2c) and UVPD (Figure 2d). Five *x* and five *c* ions were also detected in EAD, while only three and two of them were in UVPD. In both cases, a+1 and x+1 ions are detected. In CID, *y* (7) and *b* (4) ions are mainly observed. Two *a* ions are also detected with the most abundant being the a_2 fragment. 86% sequence coverage is obtained from the ETD spectrum, as z_n (n=3-8) ions and abundant c_6 and c_7 are detected (Figure 2b). Low abundance *a*/*x* and *y* ions are also produced. The charge reduced radical ion $[M+2H]^{+\bullet}$ of *m/z* 1118.48 and the singly charged $[M+H]^+$ *m/z* 1117.48 are also prominent in ETD as well as in EAD spectra.

Characteristic loss of H_3PO_4 moieties from the doubly charged precursor ion is detected at *m/z* 510.1971 in the CID, EAD and UVPD spectra. Neutral losses of water and ammonia are also observed in these spectra from the precursor and from fragment ions. In CID, all pT-containing fragments are also detected after elimination of the phosphate group: y_n (n=6-7) and b_n (n=3-5) ions. The same trend is observed in UVPD but not in EAD where the phosphate group is still intact in backbone fragments. Sequential losses of phosphate and NH_3 are also detected from the y_6 ion in CID and UVPD. Moreover, elimination of HPO_3 (79.966330 Da) is observed from the doubly charged y_7 ion in CID. Regarding fragments containing the pY residues, only the low intensity ion y_5-H_3PO_4 is detected in CID and absent in UVPD and EAD. In ETD, both phosphate groups are preserved for all backbone fragment ions containing the modification (in green in Figure 2b). The loss of H_3PO_4 group is only observed from the $[M+2H]^{+\bullet}$ radical cation in ETD and from the $[M+H]^+$ ion in EAD.

Activation of large phosphorylated ISENIS(HPO_3)ECLYGGTTLNSEK (IK19pS) and ISENISECLYGGT(HPO_3)TLNSEK (IK19pT) peptides

The CID, ETD, EAD and UVPD spectra of the triply protonated $[M+3H]^{3+}$ (*m/z* 713.3180) peptide ISENIS(H_2PO_4)ECLYGGTTLNSEK (IK19pS) and ISENISECLYGGT(H_2PO_4)TLNSEK (IK19pT) are presented in Figure S1 and Figure 3, respectively. Theoretical *m/z*, observed *m/z*, and assignments for fragment ions detected in each experiment for these peptides are summarized in Table S3 and S4.

Sequence coverage of 100% with a substantial number of fragment ions are obtained from CID spectra for the two peptides. Nearly equal numbers of *b* (20/18 for pS/pT) and *y* (43/48) ions (including PTM losses) were detected in CID for both peptides (Figure S1a and 3a). Some *a* ions are also detected in CID. Elimination of the H_3PO_4 group and NH_3, from the doubly charged precursor ion, are observed at *m/z* 1020.4831 and *m/z* 1060.9583, respectively. Neutral losses of water and ammonia are also observed in both CID spectra for several *a* and *b*/*y* fragment ions. Most of the C-terminal and N-terminal fragment ions containing the pS or pT are detected both with intact phosphate group (in green in Figure S1-3a) and after H_3PO_4/HPO_3 elimination (in red in Figure S1-3a). Exceptions are b_n-H_3PO_4 (n=6, 7, 9, 11, 15 for pS and n=14, 15 for the pT form) and doubly charged y_n-$H_3PO_4^{2+}$ (n=14-18 for both), which are detected only without the modification. Loss of HPO_3 is also observed from y_n (n=8-14) for the pT containing peptide and from y_{14} from the pS containing isomer.

The ETD experiments on these triply charged peptides mainly yield the doubly charged radical $[M+3H]^{2+\bullet}$ at *m/z* 1069.97 as well as the deprotonated species $[M+2H]^{2+}$ at *m/z* 1069.47 (Figure S1-3b) and consecutive NH_3 elimination (*m/z* 1060.96). Some low intensity singly charged radical $[M+2H]^{+\bullet}$ ions are also observed at *m/z* 2138.94 as well as $[M+2H-NH_3]^{+\bullet}$ at *m/z* 2122.93. Mainly *z* and *c* ions are detected after fragmentation of the peptide backbone (Table S3 and S4). Few *a* (4/2 for pS/pT) and *y* (1/3) are also produced for both peptides with ETD. The phosphate group is fully preserved for all fragment ions that include the initially modified site (either pT or pS) (in green in Figure S1-3b). A sequence coverage of 78% is obtained from the ETD spectra of both IK19pS and IKpT peptides.

The EAD spectra provide sequence coverage of 100% with all fragment ions types, *a*, *b*, *c*, *x*, *y*, and *z* (Figure S1-3c and Table S3-4) for both peptides. Even if a complete series of ions is not observed, the complementarity of C-terminal and N-terminal fragments allow characterization of the whole sequence. The most abundant fragment ions are mainly *a* and *c* types, and *z* ions in the

low *m/z* range. Moreover, a+1 and x+1 fragments are also observed. Additionally, neutral losses of H_2O and NH_3 from the precursor ion, the doubly charged $[M+2H]^{2+}$ and some *a*, *b*/*y* backbone fragments are observed. The charge reduced radical species $[M+3H]^{2+\bullet}$ is also observed at *m/z* 1069.9804 for both peptides. No loss of phosphate group is observed from the precursor ion nor from the fragment ions that include the initially phosphorylated site.

In UVPD, all types of fragment ions are detected with mainly *a*, *c* and *y* ions. Few x+1 (2 for the IK19pS peptide and 1 for the IK19pT peptide) are also observed and some *a*, *b*/*y* backbone fragments present a neutral loss of H_2O and NH_3, as well as the precursor ion. The loss of the H_3PO_4 group is observed (in red in Figure S1-3d) from a significant number of singly and doubly charged backbone fragment ions (13 for the pS peptide and 23 for the pT peptide) as well as from the 3+ precursor ion at *m/z* 1020.4831. Looking at the ion counts for the IK19pS peptide: a_{13}, b_n (n=8, 10, 15-18) and y_n (n=14-16) are detected both with intact phosphate group (in green in Figure 3d) and after H_3PO_4 elimination (in red in Figure S1d). Fragments b_n (n=6-7) are detected only after phosphate loss. And fragments a_n (n=10, 14-18), c_n (n=9, 12-13, 16-17), x_n (n=14-16) and y_{17} ions are observed only with the intact modification (see Table S3). For the IK19pT peptide: a_{13}-H_3PO_4, b_n-H_3PO_4 (n=14-18), x_7-H_3PO_4 (detected only without the phosphate group) and doubly and/or singly charged y_n-$H_3PO_4{}^{1+/2+}$ (n=8-18). However, a_n (n=13-18), b_n (n=14-18), c_n (n=13, 16, 17), $x_n^{1+/2+}$ (n=10-17) and $y_n^{1+/2+}$ (n=10-18) fragment ions retain the phosphate groups (in green in Figure 3d) (see Table S4). Overall, a complete sequence coverage of 100 % is obtained from the UVPD spectrum of IK19pT, and 89 % for the IK19pS peptide.

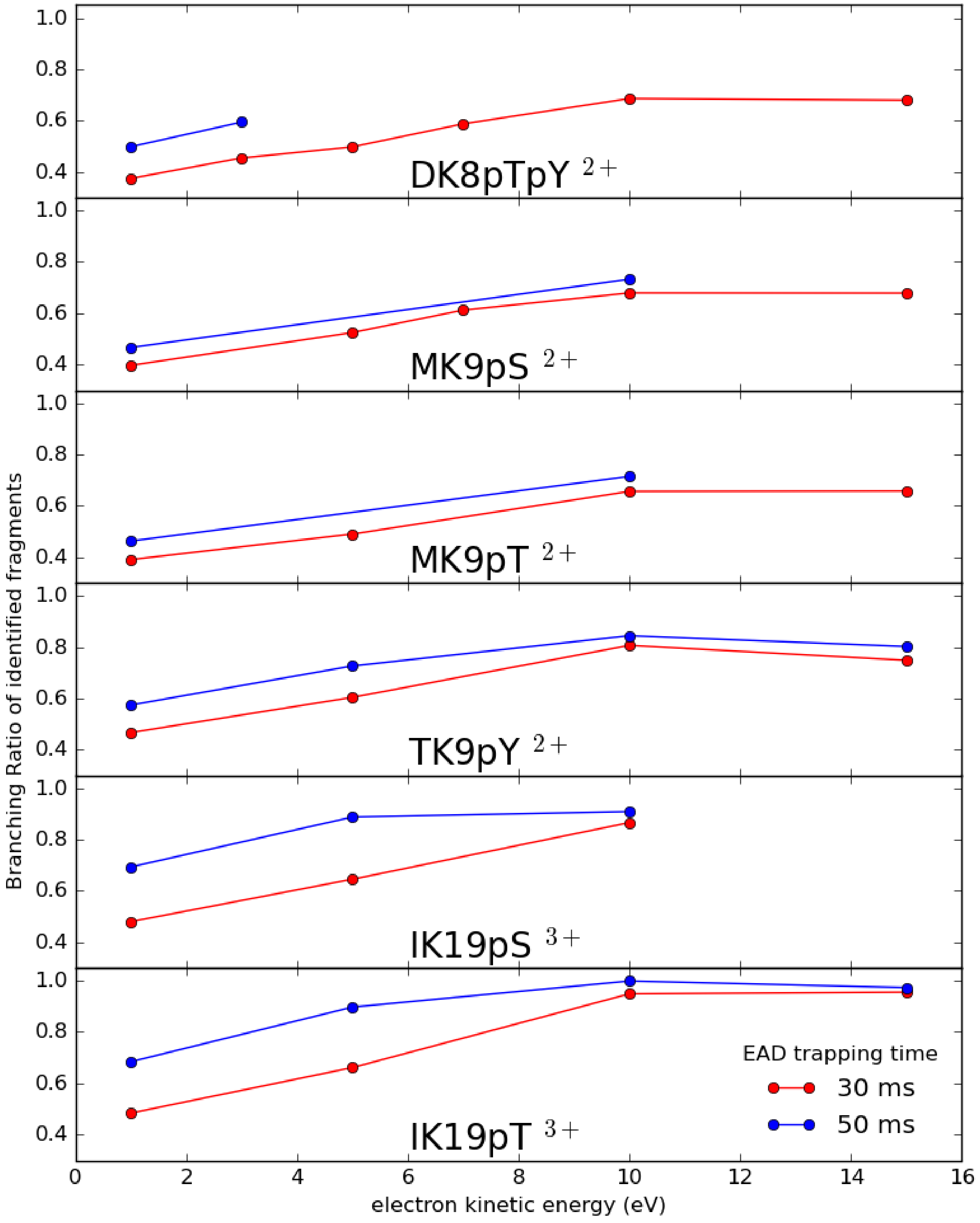

Figure S1: Branching ratios of the sum of all fragments that were used for peptide identification in the EAD spectra as a function of the electron kinetic energy and reaction time for the six synthetic phosphopeptides (doubly charged MK9pS/pT, DK8pTpY, TK9pY and triply charged IK19pS/pT).

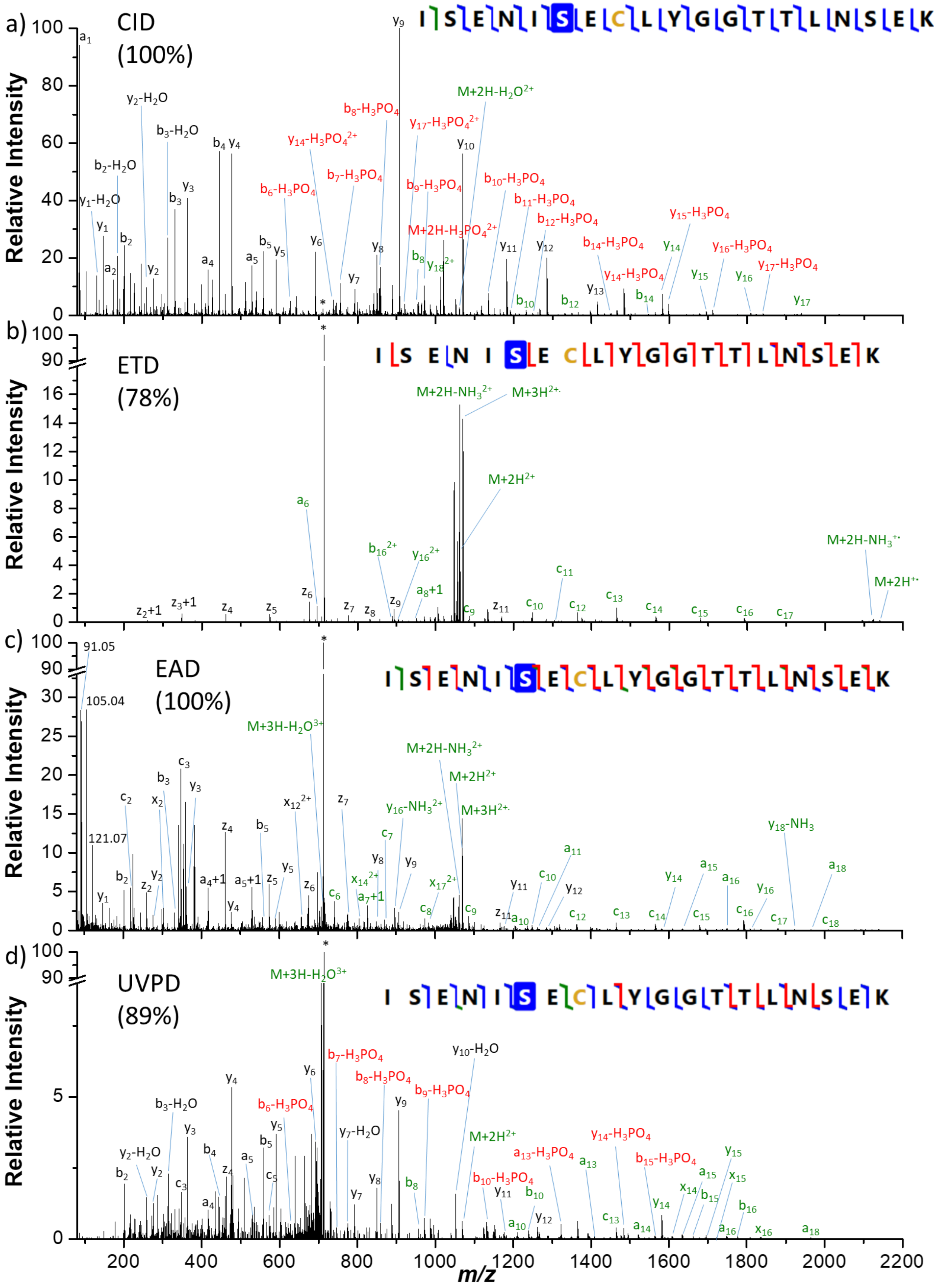

Figure S2: a) CID, b) ETD, c) EAD and d) UVPD spectra of the triply protonated [M+3H]$^{3+}$ (*m/z* 713.3180) IK19pS peptide. Precursor ions are indicated by an asterisk (*). Sequence coverages (%)

are presented between brackets. Fragments annotated in green contain the intact phosphate moieties, while fragments in red have lost the modification. Detailed assignments of the fragment ions are summarized in Table S3.

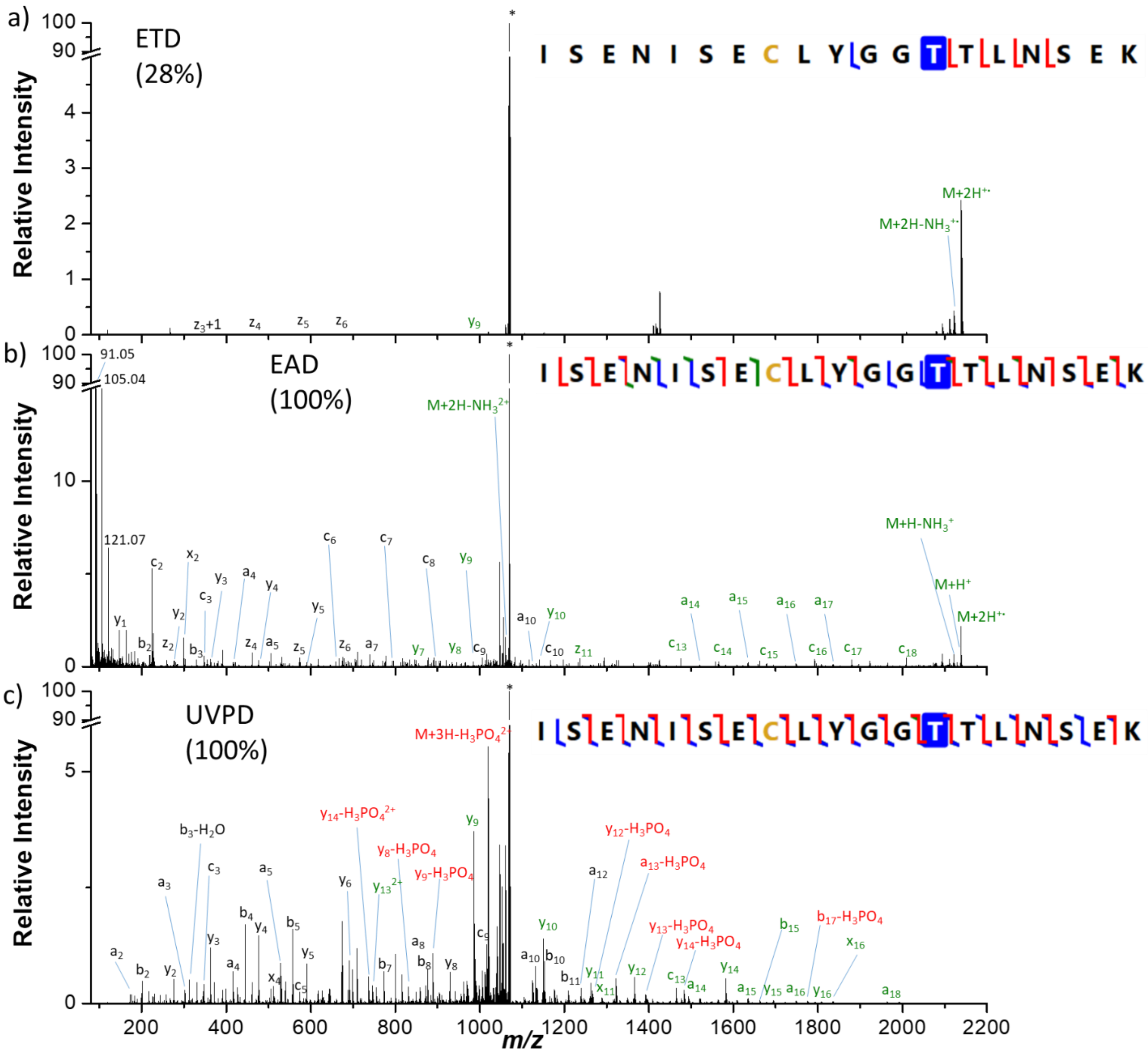

Figure S3: a) ETD, b) EAD and c) UVPD spectra of the doubly protonated $[M+2H]^{2+}$ (*m/z* 1069.4718) IK19pT peptide. Precursor ions are indicated by an asterisk (*). Sequence coverages (%) are presented between brackets. Fragments annotated in green contain the intact phosphate moieties, while fragments in red have lost the modification.

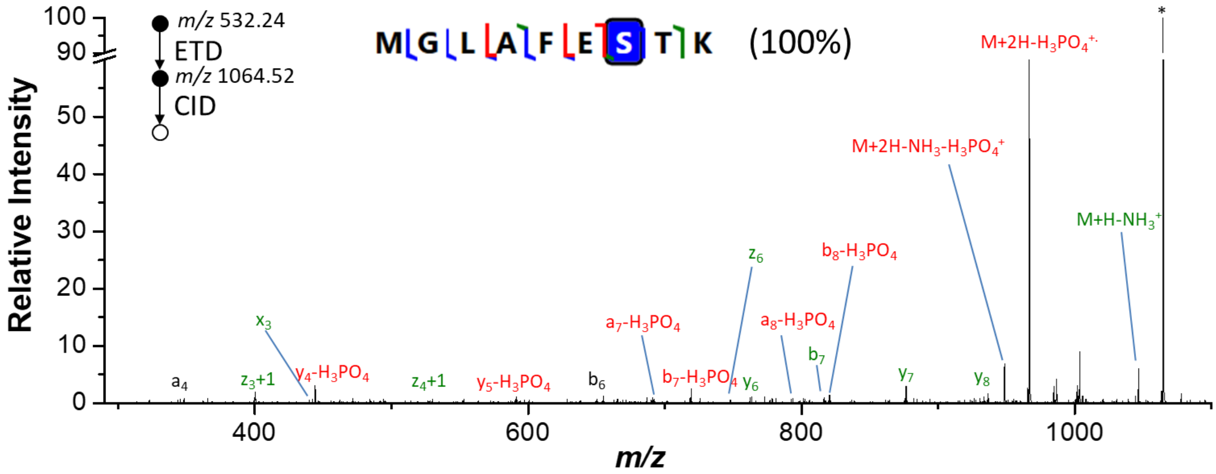

Figure S4: CID spectrum (ramping activation from NCE 15 to NCE 25) of the singly radical $[M+2H]^{+\bullet}$ (*m/z* 1064.52) MK9pS peptide formed by ETD of the doubly protonated $[M+2H]^{2+}$ (*m/z* 532.24). The precursor ions are indicated by star (*) sign. Sequence coverage (%) presented in the bracket. Fragments annotated in green contain the intact phosphate moieties, while fragments in red have lost the modification.

Table S1. PRM precursor list of the targeted phosphopeptides in the spiked digest plasma sample and summed peak areas of the 7 most intense detected fragment ions. C[+57]: cysteine carbamidomethylation; Y, S, T[+80]: phosphorylation

Peptide	*m/z*	z	KE (eV)	Reaction time (ms)	Peak Area (cps^2)
T**C[+57]**M**Y[+80]**GGITK	555.7216	2	10	30	7055.6
DP**T[+80]**NG**Y[+80]**YK	559.1872	2	10	30	1428.7
MGLAFE**S[+80]**TK	532.2301	2	10	30	23309.6
MGLAFES**T[+80]**K	532.2301	2	10	30	17355.0
ISENI**S[+80]**E**C[+57]**LYGGTTLNSEK	732.3240	3	10	30	2452.9
ISENISE**C[+57]**LYGG**T[+80]**TLNSEK	732.3240	3	10	30	2182.9

Table S2. List of fragment ion assignments with theoretical and observed *m/z* values detected in the CID, ETD, EAD and UVPD spectra of the doubly protonated $[M+2H]^{2+}$ (*m/z* 532.2301) MGLAFES(H_2PO_4)TK peptide. Errors are below ±10 ppm for all CID, EAD and UVPD assignments. The low resolution of the ion trap in ETD experiments does not allow exact masses measurements.

Assignment	Theoretical *m/z*	CID Observed *m/z*	ETD Observed *m/z*	EAD Observed *m/z*	UVPD Observed *m/z*
$(a_1)^+$	104.0529	104.0525	-	104.0533	104.0529
$(a_2)^+$	161.0743	161.0738	-	161.0753	161.0743
$(a_3)^+$	274.1584	274.1574	-	-	-
$(a_3+1)^+$	275.1662	-	-	275.1639	275.1665
$(a_4)^+$	345.1955	345.1937	-	-	345.1955
$(a_4+1)^+$	346.2033	-	-	346.2048	-
$(a_5)^+$	492.2639	492.2615	-	-	-
$(a_6+1)^+$	622.3143	-	-	622.3144	622.3149
$(a_8-1)^+$	888.3447	-	-	888.3437	-
$(b_2)^+$	189.0692	189.0687	-	189.0702	189.0693
$(b_2-H_2O)^+$	171.0587	171.0574	-	-	171.0575
$(b_3)^+$	302.1533	302.1523	-	302.1554	302.1533
$(b_4)^+$	373.1904	373.1891	-	373.1936	373.1902
$(b_5)^+$	520.2588	520.2542	-	520.2581	520.2581
$(b_7)^+$	816.2998	-	-	816.3057	-
$(c_2)^+$	206.0958	206.0957	-	-	206.0957
$(c_3)^+$	319.1798	-	-	319.182	-
$(c_4-1)^+$	389.2092	389.2116	-	-	389.2109
$(c_6)^+$	666.328	-	666.36	-	-
$(c_7)^+$	833.3263	-	833.40	833.3244	-
$(c_8)^+$	934.374	-	934.40	-	-
$(M+2H)^{2+}$	532.2301	532.2288	532.24	532.2261	532.2305
$(M+2H-H_3PO_4)^{2+}$	483.2417	483.2403	-	483.2418	483.2419
$(M+2H-NH_3)^{2+}$	523.7168	-	-	523.7189	523.7189
$(M+H)^+$	1063.453	-	1063.56	1063.4577	-
$(M+2H)^{+\bullet}$	1064.5678	-	1064.52	1064.5663	-
$(M+H-H_3PO_4)^+$	965.4761	-	-	965.4812	-
$(w_{a4})^+$	469.1694	469.1694	-	469.173	469.1694
$(w_{a9})^+$	986.4231	-	-	986.4266	986.4228
$(x_3)^+$	441.1381	-	-	441.1419	441.1377
$(x_4+1)^+$	571.1885	-	-	-	571.1883
$(x_5)^+$	717.2491	-	-	-	717.2492
$(x_5+1)^+$	718.2569	-	-	718.2508	-
$(x_6)^+$	788.2862	-	-	788.2865	-
$(y_1)^+$	147.1128	-	-	-	147.1128

$(y_2)^+$	248.1605	248.1598	-	248.1624	248.1605
$(y_2\text{-}H_2O)^+$	230.1499	230.149	-	-	-
$(y_3)^+$	415.1588	415.1577	-	415.1625	415.1589
$(y_3\text{-}H_3PO_4)^+$	317.1819	317.1811	-	317.1834	317.182
$(y_4)^+$	544.2014	544.1995	-	544.2014	544.2011
$(y_4\text{-}H_2O)^+$	526.1909	526.1891	-	-	-
$(y_4\text{-}H_3PO_4)^+$	446.2245	446.22374	-	446.2281	446.2245
$(y_5)^+$	691.2699	691.2679	691.32	691.2709	691.27
$(y_5\text{-}H_2O\text{-}H_3PO_4)^+$	575.2824	575.2801	-	-	-
$(y_5\text{-}H_3PO_4)^+$	593.293	593.2914	-	593.292	593.2929
$(y_6)^+$	762.307	762.305	762.36	762.3073	762.3068
$(y_6\text{-}H_2O\text{-}H_3PO_4)^+$	646.3195	646.3167	-	-	-
$(y_6\text{-}H_3PO_4)^+$	664.3301	664.3282	-	664.3305	664.3302
$(y_7)^+$	875.391	875.3876	875.48	875.3909	875.3919
$(y_7\text{-}H_3PO_4)^+$	777.4141	777.4113	-	-	777.4147
$(y_8)^+$	932.4125	932.4096	932.48	932.4151	932.412
$(y_8\text{-}H_2O\text{-}H_3PO_4)^+$	816.425	816.4215	-	-	-
$(y_8\text{-}H_3PO_4)^+$	834.4356	834.4329	-	-	834.43565
$(z_1)^+$	131.0941	-	-	131.0935	131.0941
$(z_2)^+$	232.1418	-	-	232.1409	232.1418
$(z_3)^+$	399.1401	-	-	399.1407	399.1401
$(z_3+1)^+$	400.1479	-	400.24	-	-
$(z_4)^+$	528.1827	-	-	528.1834	528.183
$(z_5)^+$	675.2511	-	675.32	675.2515	-
$(z_6)^+$	746.2882	-	746.36	746.2882	746.2898
$(z_7)^+$	859.3723	-	859.44	859.3707	-
$(z_7+1)^{2+}$	430.1898	-	-	430.1882	-
$(z_8)^+$	916.3938	-	916.48	916.3933	-
$(z_8+1)^{2+}$	459.2047	-	-	-	459.205
$(z_9)^+$	1047.4343	-	1047.52	-	-

Table S3. List of fragment ion assignments with theoretical and observed *m/z* values detected in the CID, ETD, EAD and UVPD spectra of the doubly protonated $[M+2H]^{2+}$ (*m/z* 559.1872) DPT(H_2PO_4)NGY(H_2PO_4)YK peptide. Errors are below ±10 ppm for all CID, EAD and UVPD assignments. The low resolution of the ion trap in ETD experiments does not allow exact masses measurements.

Assignment	Theoretical *m/z*	CID Observed *m/z*	ETD Observed *m/z*	EAD Observed *m/z*	UVPD Observed *m/z*
$(a_1)^+$	88.0393	88.03907	-	88.039	88.0395
$(a_2)^+$	185.0921	185.0915	-	185.0922	185.0922
$(a_3)^+$	366.1061	-	-	366.1065	366.1062
$(a_4)^+$	481.1568	-	-	-	-
$(a_6+1)^+$	781.2079	-	-	781.2095	781.2076
$(a_7)^+$	943.2635	-	-	943.2667	943.2632
$(a_7+1)^+$	944.36	-	944.36	-	-
$(b_2)^+$	213.087	213.0861	-	213.0872	213.0871
$(b_2\text{-}H_2O)^+$	195.0764	195.0757	-	-	195.0765
$(b_3)^+$	394.101	394.0997	-	394.1028	394.1011
$(b_3\text{-}H_3PO_4)^+$	296.1241	296.1232	-	-	296.1241
$(b_4)^+$	508.1439	508.1396	-	508.1467	508.1442
$(b_4\text{-}H_3PO_4)^{2+}$	410.167	410.1654	-	-	-
$(b_5)^+$	565.1654	565.1632	-	-	565.1657
$(b_5\text{-}H_3PO_4)^+$	467.1885	467.1846	-	-	-
$(b_6)^+$	808.195	-	-	808.1963	-
$(b_6\text{-}NH_3)^+$	791.1685	-	-	791.171	-
$(b_7)^+$	971.2584	-	-	971.2619	971.2569
$(b_7\text{-}H_3PO_4)^+$	873.2815	-	-	-	873.2821
$(c_3)^+$	411.1275	-	-	411.1299	-
$(c_3\text{-}1)^+$	410.1197	-	-	-	410.1171
$(c_4)^+$	525.1705	-	-	525.1724	-
$(c_5)^+$	582.1919	-	-	582.1922	-
$(c_6)^+$	825.1705	-	825.32	825.1734	825.172
$(c_7)^+$	988.2849	-	988.36	988.2893	-
$(M+2H)^{2+}$	559.1856	559.1903	559.24	559.1903	559.1855
$(M+2H\text{-}H_2O)^{2+}$	550.1804	550.1768	-	550.1809	550.1757
$(M+2H\text{-}H_3PO_4)^{+\bullet}$	1020.48	-	1020.48	-	-
$(M+2H\text{-}H_3PO_4)^{2+}$	510.1972	510.1955	-	510.2015	510.1973
$(M+2H\text{-}NH_3)^+$	1100.3374	-	-	1100.3448	-
$(M+H)^+$	1117.48	-	1117.48	1117.3706	-
$(M+2H)^{+\bullet}$	1118.3747	-	1118.48	1118.3763	-
$(M+H\text{-}H_3PO_4)^+$	1019.39	-	-	1019.391	-
$(x_2+1)^+$	337.1632	-	-	337.1646	337.1633
$(x_4)^+$	636.2065	-	-	636.2054	-
$(x_4+1)^+$	637.2164	-	-	637.2121	-
$(x_5)^+$	750.25	-	-	750.2514	750.25

$(x_5+1)^+$	751.2578	-	751.27	-	-
$(x_5+1)^+$	932.2718	-	-	932.2748	932.2702
$(x_6-1)^+$	930.2562	-	-	930.2479	-
$(y_1)^+$	147.1128	147.1131	-	147.1129	147.1129
$(y_1-H_2O)^+$	129.1022	129.1022	-	-	129.1023
$(y_1-NH_3)^+$	130.0863	130.0868	-	-	-
$(y_2)^+$	310.1761	310.1753	-	310.1776	310.1762
$(y_2-H_2O)^+$	292.1656	292.1644	-	292.1629	292.1639
$(y_3)^+$	553.2058	553.2017	-	553.2009	553.2065
$(y_4)^+$	610.2273	610.2252	-	610.2282	610.2273
$(y_5)^+$	724.2702	724.2678	-	724.2709	724.2703
$(y_5-1)^+$	723.2624	-	723.32	-	-
$(y_5-H_3PO_4)^+$	626.2933	626.2892	-	-	-
$(y_5-NH_3)^+$	707.2436	707.2408	-	707.2445	707.2435
$(y_6)^+$	905.2842	905.2894	905.36	905.2862	905.2815
$(y_6)^{2+}$	453.1457	-	-	-	453.1474
$(y_6-H_3PO_4)^+$	807.3073	807.3045	-	-	807.3072
$(y_6-H_3PO_4)^{2+}$	404.1575	404.1559	-	-	404.1575
$(y_6-NH_3-H_3PO_4)^+$	790.2808	790.2811	-	-	790.28
$(y_6-NH_3-H_3PO_4)^{2+}$	395.6443	395.6429	-	-	395.6439
$(y_7)^+$	1002.337	-	-	1002.3356	-
$(y_7)^{2+}$	501.6724	501.6752	-	501.6767	501.67
$(y_7+1)^+$	1001.3297	-	-	1001.3254	-
$(y_7-H_2O)^{2+}$	492.6671	492.6717	-	-	492.6692
$(y_7-H_3PO_4)^+$	904.36	904.3601	-	-	-
$(y_7-H_3PO_4)^{2+}$	452.6839	452.6827	-	-	452.6836
$(y_7-HPO_3)^{2+}$	461.6731	461.6768	-	-	-
$(z_1)^+$	131.0941	-	-	131.0936	-
$(z_2)^+$	294.1574	-	-	294.1578	294.1578
$(z_3)^+$	537.187	-	537.24	537.1901	-
$(z_4)^+$	594.2085	-	594.28	594.2092	-
$(z_5)^+$	708.2515	-	708.32	708.2523	-
$(z_6)^+$	889.2655	-	889.36	889.2664	889.2648
$(z_7)^{2+}$	493.5039	-	-	493.4999	-
$(z_8)^+$	1101.44	-	1101.44	1100.3448	-

Table S4. List of fragment ion assignments with theoretical and observed *m/z* values detected the CID, ETD, EAD and UVPD spectra of the triply protonated $[M+3H]^{3+}$ (*m/z* 713.3180) ISENIS(H_2PO_4)ECLYGGTTLNSEK peptide. Errors are below ±10 ppm for all CID, EAD and UVPD assignments. The low resolution of the ion trap in ETD experiments does not allow exact masses measurements.

Assignment	Theoretical *m/z*	CID Observed *m/z*	ETD Observed *m/z*	EAD Observed *m/z*	UVPD Observed *m/z*
$(a_1)^+$	86.0964	86.0960	-	86.0971	-
$(a_2)^+$	173.1285	173.127	-	173.1302	173.1285
$(a_3)^+$	302.171	-	-	302.1729	302.1711
$(a_4)^+$	416.214	416.2106	-	-	416.214
$(a_4+1)^+$	417.2199	-	-	417.2233	-
$(a_4-NH_3)^+$	399.1874	399.1846	-	-	399.1874
$(a_5)^+$	529.298	529.2932	-	529.3021	529.2981
$(a_5+1)^+$	530.3079	-	-	530.3103	-
$(a_5-NH_3)^+$	512.2715	512.2683	-	-	-
$(a_6)^+$	696.2964	-	696.3	696.2917	-
$(a_7)^+$	825.339	-	-	825.3463	-
$(a_7-1)^+$	826.3489	-	-	826.3521	-
$(a_8)^+$	928.3482	-	-	928.355	-
$(a_8+1)^+$	924.356	-	929.4	-	-
$(a_{10})^+$	1204.4956	-	-	1204.4965	1204.498
$(a_{11})^+$	1261.517	-	-	1261.5287	-
$(a_{13})^+$	1419.5862	-	-	-	1419.593
$(a_{13}-H_3PO_4)^+$	1321.6093	-	-	-	1321.6185
$(a_{14})^+$	1520.6339	-	-	-	1520.6275
$(a_{15})^+$	1633.7179	-	-	1633.7175	-
$(a_{15})^+$	1633.7179	-	-	-	1633.7191
$(a_{16})^+$	1747.7608	-	-	1747.7571	1747.7618
$(a_{16}+1)^+$	1748.7686	-	1748.78	-	-
$(a_{18})^+$	1963.8355	-	-	1963.8308	1963.8353
$(b_2)^+$	201.1234	201.1218	-	201.1253	201.1235
$(b_2-H_2O)^+$	183.1128	183.1114	-	-	-
$(b_3)^+$	330.166	330.1638	-	-	-
$(b_3-H_2O)^+$	312.1554	312.1536	-	-	312.1556
$(b_4)^+$	444.2089	444.2054	-	444.2125	444.209
$(b_4-H_2O)^+$	426.1983	426.1945	-	-	426.1984
$(b_5)^+$	557.293	557.2879	-	557.2958	557.2935
$(b_5-H_2O)^+$	539.2824	539.2771	-	-	-
$(b_6-H_3PO_4)^+$	626.3144	626.3087	-	-	626.3145
$(b_7)^+$	853.3339	-	-	853.3395	-
$(b_7-H_3PO_4)^+$	755.357	755.3501	-	-	755.3571
$(b_8)^+$	956.3431	956.3336	-	956.3453	956.3437
$(b_8-H_3PO_4)^+$	858.3661	858.359	-	-	858.3674

$(b_9\text{-}H_3PO_4)^+$	971.4503	971.4451	-	-	971.4525
$(b_{17}\text{-}H_3PO_4)^+$	1764.8109	-	-	-	1764.7936
$(b_{18})^+$	1991.8304	-	-	-	1991.8331
$(b_{18}\text{-}H_3PO_4)^+$	1893.8535	-	-	-	1893.8531
$(c_1)^+$	131.1179	-	-	131.119	-
$(c_2)^+$	218.1499	-	-	218.1513	-
$(c_3)^+$	347.1925	-	-	347.1934	347.1898
$(c_5)^+$	574.3195	-	-	-	574.3201
$(c_6)^+$	741.3179	-	-	741.3225	-
$(c_7)^+$	870.3605	-	-	870.3649	-
$(c_8)^+$	973.3696	-	-	973.3759	-
$(c_9)^+$	1086.4537	-	1086.45	1086.4572	1086.4529
$(c_{10})^+$	1249.517	-	1249.51	1249.5215	-
$(c_{11})^+$	1306.5385	-	1306.53	1306.5439	-
$(c_{12})^+$	1363.56	-	1363.56	1363.5622	-
$(c_{12}+2)^+$	1365.5667	-	-	-	1365.5774
$(c_{13})^+$	1464.6076	-	1464.61	1464.6101	1464.6086
$(c_{14})^+$	1565.6553	-	1565.65	1565.6506	-
$(c_{15})^+$	1678.7394	-	1678.74	1678.7362	-
$(c_{16})^+$	1792.7823	-	1792.78	1792.7788	1792.7781
$(c_{17})^+$	1879.8143	-	1879.81	1879.8096	1879.8168
$(c_{18})^+$	2008.8569	-	2008.85	2008.8568	-
$(M+3H)^{3+}$	713.318	713.3178	713.55	713.3167	713.3157
$(M+3H\text{-}H_2O)^{3+}$	707.315	-	-	707.3219	707.3131
$(M+2H)^+$	1069.4716	-	1069.47	1069.4793	1069.4713
$(M+3H)^{2+\bullet}$	1069.9755	-	1069.97	1069.9804	-
$(M+2H\text{-}H_2O)^+$	1060.4661	1060.4561	-	-	-
$(M+2H\text{-}H_3PO_4)^{2+}$	1020.4831	1020.4733	-	-	1020.4833
$(M+2H\text{-}NH_3)^+$	1060.9583	-	1060.97	1060.9625	-
$(M+2H\text{-}NH_3)^+$	2121.9432	-	2122.93	-	-
$(M+H)^+$	2137.9359	-	2137.92	-	-
$(M+2H)^{+\bullet}$	2138.9432	-	2138.94	-	-
$(w_{a19})^{2+}$	1046.9427	-	1046.95	-	-
$(x_1)^+$	173.0921	-	-	173.0937	-
$(x_2)^+$	302.1347	-	-	302.1371	-
$(x_3)^+$	389.1667	-	-	389.1703	-
$(x_4)^+$	503.2096	-	-	503.2117	-
$(x_4+1)^+$	504.2195	-	-	504.2212	-
$(x_4+2)^+$	505.2285	-	-	505.2287	-
$(x_6)^+$	717.3414	-	-	-	717.342
$(x_{10})^{2+}$	548.2513	-	-	548.2547	-
$(x_{10}+1)^+$	1096.5052	-	-	1096.5036	-
$(x_{11}+1)^+$	1209.5871	-	-		1209.5866
$(x_{12})^{2+}$	656.2979	-	-	656.3015	-
$(x_{14})^+$	1607.6295	-	-		1607.6289

$(x_{14})^{2+}$	804.3184	-	-	804.3216	-
$(x_{15})^{+}$	1720.7136	-	-	1720.7228	1720.6999
$(x_{15})^{2+}$	860.8604	-	-	860.868	-
$(x_{16})^{+}$	1834.7565	-	-	-	1834.7718
$(x_{16})^{2+}$	917.8819	-	-	917.8878	-
$(x_{17})^{2+}$	982.4032	-	-	982.4112	-
$(y_1\text{-}H_2O)^{+}$	129.1022	129.1014	-	-	-
$(y_1\text{-}NH_3)^{+}$	130.0863	130.0854	-	-	-
$(y_2)^{+}$	147.1128	147.1114	-	147.1137	-
$(y_2)^{+}$	276.1554	276.1536	-	276.1566	276.1554
$(y_2\text{-}H_2O)^{+}$	258.1448	258.1426	-	-	258.1449
$(y_3)^{+}$	363.1874	363.1841	-	363.1904	363.1873
$(y_3\text{-}H_2O)^{+}$	345.1769	345.1739	-	-	345.174
$(y_4)^{+}$	477.2304	477.2265	-	477.2344	477.2304
$(y_4\text{-}H_2O)^{+}$	459.2198	459.2153	-	-	-
$(y_5)^{+}$	590.3144	590.3089	-	590.3177	590.3146
$(y_6)^{+}$	691.3621	691.3553	-	691.3661	691.3628
$(y_6\text{-}H_2O)^{+}$	673.3515	673.3451	-	673.3492	-
$(y_6\text{-}NH_3)^{+}$	674.3355	-	-	674.3395	674.3353
$(y_7)^{+}$	792.4098	792.4021	-	792.4133	792.4098
$(y_7\text{-}H_2O)^{+}$	774.3992	774.3921	-	774.3925	774.4039
$(y_7\text{-}NH_3)^{+}$	775.3832	-	-	775.3866	-
$(y_8)^{+}$	849.4312	849.4239	-	849.4351	849.4323
$(y_8\text{-}1)^{+}$	848.4213	-	-	848.4206	-
$(y_8\text{-}H_2O)^{+}$	831.4207	831.4128	-	-	-
$(y_9)^{+}$	906.4527	906.4451	-	906.4561	906.4532
$(y_9)^{2+}$	453.73	-	-	453.7336	-
$(y_9\text{-}H_2O)^{+}$	888.4421	888.4337	-	-	888.4432
$(y_{10})^{+}$	1069.516	1069.5055	-	-	-
$(y_{10})^{2+}$	535.2617	-	-	535.2657	535.262
$(y_{10}\text{-}H_2O)^{+}$	1051.5055	1051.4956	-	1051.4998	1051.4981
$(y_{11})^{+}$	1182.6001	1182.5891	-	1182.5986	1182.6016
$(y_{11}\text{-}H_2O)^{+}$	1164.5895	1164.5786	-	-	-
$(y_{12})^{+}$	1285.6093	1285.5966	-	1285.6088	1285.609
$(y_{12}\text{-}H_2O)^{+}$	1267.5987	1267.5861	-	-	-
$(y_{13})^{+}$	1414.6519	1414.6379	-	1414.655	-
$(y_{13}\text{-}H_2O)^{+}$	1396.6413	1396.6279	-	-	-
$(y_{14})^{+}$	1581.6502	1581.6372	-	1581.6484	1581.6519
$(y_{14}\text{-}H_2O)^{+}$	1563.6397	1563.6251	-	-	-
$(y_{14}\text{-}H_3PO_4)^{+}$	1483.6733	1483.6596	-	-	1483.6741
$(y_{14}\text{-}H_3PO_4)^{2+}$	742.3403	742.3351	-	-	-
$(y_{14}\text{-}HPO_3)^{+}$	1501.6839	1501.6688	-	-	-
$(y_{15})^{+}$	1694.7343	1694.7233	-	1694.7302	1694.7301
$(y_{15}\text{-}H_2O)^{+}$	1676.7237	1676.7244	-	-	-
$(y_{15}\text{-}H_3PO_4)^{+}$	1596.7574	1596.7419	-	-	1596.7551

$(y_{15}\text{-}H_3PO_4)^{2+}$	798.8823	798.8744	-	-	-
$(y_{15}\text{-}NH_3)^{2+}$	839.3575	-	-	839.3631	-
$(y_{16})^{+}$	1808.7772	1808.7571	-	1808.7753	1808.7737
$(y_{16})^{2+}$	904.8925	-	904.89	-	-
$(y_{16}\text{-}H_3PO_4)^{+}$	1710.8003	1710.7834	-	-	1710.8013
$(y_{16}\text{-}H_3PO_4)^{2+}$	855.9038	855.8958	-	-	-
$(y_{16}\text{-}NH_3)^{2+}$	896.379	-	-	896.3822	-
$(y_{17})^{+}$	1937.8198	1937.8273	-	-	1937.8186
$(y_{17}\text{-}H_3PO_4)^{+}$	1839.8429	1839.8246	-	-	-
$(y_{17}\text{-}H_3PO_4)^{2+}$	920.4251	920.4163	-	-	-
$(y_{17}\text{-}NH_3)^{+}$	1920.7933	-	-	1920.7917	-
$(y_{18})^{2+}$	1012.9296	1012.9229	-	1012.939	-
$(y_{18}\text{-}H_3PO_4)^{2+}$	963.9411	963.933	-	-	-
$(y_{18}\text{-}NH_3)^{2+}$	960.9003	-	-	960.9062	-
$(z_{1})^{+}$	131.0941	-	-	131.0946	-
$(z_{2})^{+}$	260.1367	-	-	260.1387	-
$(z_{2}+1)^{+}$	261.1445	-	261.14	-	-
$(z_{3})^{+}$	347.1667	-	-	347.1642	-
$(z_{3}+1)^{+}$	348.1745	-	348.18	-	-
$(z_{4})^{+}$	461.2116	-	461.21	461.2148	461.2116
$(z_{5})^{+}$	574.2957	-	574.3	574.3006	-
$(z_{6})^{+}$	675.3434	-	675.34	675.3476	-
$(z_{7})^{+}$	776.391	-	776.39	776.3946	-
$(z_{8})^{+}$	833.4125	-	833.41	833.4152	-
$(z_{9})^{+}$	890.434	-	890.43	890.4372	-
$(z_{9}+3)^{+}$	893.4574	-	893.4	-	-
$(z_{11})^{+}$	1166.6048	-	1166.58	1166.5825	-
$(z_{11}+3)^{+}$	1169.58	-	1169.58	-	-
$(z_{13})^{+}$	1398.6332	-	1398.63	-	-
$(z_{17}+1)^{+}$	1922.8089	-	1922.81	-	-
$(z_{17}+2)^{2+}$	962.4122	-	962.47	-	-
$(z_{18})^{2+}$	1004.9202	-	-	1004.9299	-

Table S5. List of fragment ion assignments with theoretical and observed *m/z* values detected in the CID, ETD, EAD and UVPD spectra of the triply protonated $[M+3H]^{3+}$ (*m/z* 713.3180) ISENISECLYGGT(H_2PO_4)TLNSEK peptide Errors are below ±10 ppm for all CID, EAD and UVPD assignments. The low resolution of the ion trap in ETD experiments does not allow exact masses measurements.

Assignment	Theoretical *m/z*	CID Observed *m/z*	ETD Observed *m/z*	EAD Observed *m/z*	UVPD Observed *m/z*
$(a_1)^+$	86.0964	86.0964	-	86.0967	-
$(a_2)^+$	173.1285	173.1277	-	173.1294	173.1283
$(a_3)^+$	302.171	-	-	302.1725	302.1705
$(a_4)^+$	416.214	416.2108	-	-	416.214
$(a_4+1)^+$	417.2199	-	-	417.2239	-
$(a_4-NH_3)^+$	399.1874	399.1844	-	-	399.1874
$(a_5)^+$	529.298	529.2947	-	529.2998	529.2981
$(a_5+1)^+$	530.3079	-	-	530.3103	-
$(a_5-NH_3)^+$	512.2715	512.2691	-	-	512.2718
$(a_6)^+$	616.3301	-	-	616.3299	-
$(a_6-NH_3)^+$	599.3035	-	-	-	599.3064
$(a_7)^+$	745.3727	745.3729	-	-	745.3739
$(a_7+1)^+$	746.3817	-	746.38	746.3856	-
$(a_7-NH_3)^+$	728.3461	728.3448	-	-	-
$(a_8)^+$	848.3818	-	-	-	848.3851
$(a_9)^+$	961.4659	-	-	961.4634	961.4698
$(a_9+1)^+$	962.4758	-	-	962.4758	-
$(a_9-NH_3)^+$	944.4394	-	-	-	944.4363
$(a_{10})^+$	1124.5292	-	-	1124.5311	1124.5286
$(a_{10}+1)^+$	1125.537	-	1125.54	-	-
$(a_{11})^+$	1181.5507	-	-	1181.56	-
$(a_{12})^+$	1238.5722	-	-	-	1238.5755
$(a_{13})^+$	1419.5862	-	-	1419.6005	1419.593
$(a_{13}-H_3PO_4)^+$	1321.6093	-	-	-	1321.6167
$(a_{14})^+$	1520.6339	-	-	1520.619	1520.6275
$(a_{15})^+$	1633.7179	-	-	1633.728	1633.7232
$(a_{15}-H_3PO_4)^+$	1535.741	-	-	-	1535.7414
$(a_{16})^+$	1747.7608	-	-	-	1747.7711
$(a_{17})^+$	1834.7929	-	-	1834.791	-
$(a_{18})^+$	1963.8355	-	-	1963.8296	1963.8358
$(b_2)^+$	201.1234	201.1218	-	201.1254	201.1231
$(b_2-H_2O)^+$	183.1128	183.1113	-	-	-
$(b_3)^+$	330.166	330.1639	-	330.1675	-
$(b_3-H_2O)^+$	312.1554	312.1525	-	-	312.155
$(b_4)^+$	444.2089	444.2057	-	444.211	444.209
$(b_4-H_2O)^+$	426.1983	426.1949	-	-	426.1984

$(b_5)^+$	557.293	557.2891	-	557.2947	557.293
$(b_5\text{-}H_2O)^+$	539.2824	539.279	-	-	-
$(b_5\text{-}NH_3)^+$	540.2664	-	-	-	540.2665
$(b_6)^+$	644.325	644.3186	-	-	-
$(b_6\text{-}H_2O)^+$	626.3144	626.3099	-	-	626.3145
$(b_7)^+$	773.3676	-	-	-	773.367
$(b_7\text{-}H_2O)^+$	755.357	755.3514	-	-	755.3571
$(b_8)^+$	876.3768	876.3685	-	-	876.3781
$(b_8\text{-}H_2O)^+$	858.3661	858.359	-	-	858.3662
$(b_9\text{-}H_2O)^+$	971.4503	971.4454	-	-	971.453
$(b_{10})^+$	1152.5242	-	-	1152.5257	1152.5272
$(b_{10}\text{-}H_2O)^+$	1134.5136	1134.5018	-	-	-
$(b_{11})^+$	1209.5456	-	-	-	1209.5507
$(b_{11}\text{-}H_2O)^+$	1191.5351	1191.5224	-	-	-
$(b_{12})^+$	1266.5671	-	-	-	1266.5635
$(b_{14})^+$	1548.6288	-	-	-	1548.6271
$(b_{14}\text{-}H_3PO_4)^+$	1450.6519	1450.6393	-	-	1450.6645
$(b_{15})^+$	1661.7128	-	-	-	1661.7158
$(b_{15}\text{-}H_3PO_4)^+$	1563.7359	1563.7208	-	-	1563.7391
$(b_{16})^+$	1775.7558	-	-	-	1775.752
$(b_{16}\text{-}H_3PO_4)^+$	1677.7789	-	-	-	1677.7764
$(b_{17})^+$	1862.7878	-	-	-	1862.7821
$(b_{17}\text{-}H_3PO_4)^+$	1764.8109	-	-	-	1764.7961
$(b_{18})^+$	1991.8304	-	-	-	1991.8362
$(b_{18}\text{-}H_3PO_4)^+$	1893.8535	-	-	-	1893.842
$(c_1)^+$	131.1179	-	-	131.1192	-
$(c_2)^+$	218.1499	-	-	218.1511	-
$(c_3)^+$	347.1925	-	-	347.1924	347.1898
$(c_5)^+$	574.3195	-	-	-	574.3201
$(c_6)^+$	661.3515	-	661.35	661.3561	-
$(c_8)^+$	893.4033	-	893.4	893.4048	-
$(c_9)^+$	1006.4874	-	1006.49	1006.4898	1006.4889
$(c_{10})^+$	1169.5507	-	1169.55	1169.5516	-
$(c_{11})^+$	1226.5722	-	1226.57	1226.5736	-
$(c_{12})^+$	1283.5936	-	1283.59	1283.5951	-
$(c_{13})^+$	1464.6076	-	1464.61	1464.6091	1464.6086
$(c_{14})^+$	1565.6553	-	1565.66	1565.6538	-
$(c_{15})^+$	1678.7394	-	1678.74	1678.737	-
$(c_{16})^+$	1792.7823	-	1792.78	1792.7785	1792.7899
$(c_{17})^+$	1879.8143	-	1879.81	1879.8126	1879.8061
$(c_{18})^+$	2008.8569	-	2008.85	2008.8502	-
$(M+3H)^{3+}$	713.318	713.3176	713.55	713.3201	713.3193
$(M+3H\text{-}H_2O)^{3+}$	707.315			707.3212	707.3137
$(M+3H\text{-}H_3PO_4)^{3+}$	680.659				680.6581
$(M+2H)^+$	1069.4716	-	1069.47	1069.4778	1069.4752

$(M+3H)^{2+\bullet}$	1069.9755	-	1069.97	1069.981	-
$(M+2H-H_2O)^+$	1060.4661	1060.4563	-	-	-
$(M+2H-H_3PO_4)^{2+}$	1020.4831	1020.475	-	-	1020.4861
$(M+2H-NH_3)^{2+}$	1060.9583	-	1060.97	1060.9625	1060.9656
$(M+H)^+$	2137.9359	-	2137.93		
$(M+2H)^{+\bullet}$	2138.9432	-	2138.94	-	-
$(M+2H-NH_3)^+$	2121.9432	-	2122.93	-	-
$(w_{a18})^{2+}$	996.4188	-	-		996.4182
$(x_1)^+$	173.0921	-	-	173.0935	-
$(x_2)^+$	302.1347	-	-	302.1379	-
$(x_3)^+$	389.1667	-	-	389.1702	-
$(x_4)^+$	503.2096	-	-	503.2117	503.21
$(x_4+1)^+$	504.2195	-	-	504.2191	-
$(x_4+2)^+$	505.2285	-	-	505.2284	-
$(x_5+1)^+$	617.3015	-	-	-	617.3057
$(x_6)^+$	717.3414	-	-	717.3426	-
$(x_7-H_3PO_4)^+$	800.3785	-	-	-	800.3817
$(x_8)^+$	955.3768	-	-	955.3833	-
$(x_{11})^+$	1288.5457	-	-	-	1288.5468
$(x_{12})^+$	1391.5549	-	-	1391.5672	1391.5544
$(x_{13})^{2+}$	760.8024	-	-	760.809	-
$(x_{14})^+$	1607.6295	-	-	-	1607.6289
$(x_{14})^{2+}$	804.3184	-	-	804.3201	-
$(x_{15})^+$	1720.7136	-	-	-	1720.7181
$(x_{15})^{2+}$	860.8604	-	-	860.8674	-
$(x_{16})^+$	1834.7565	-	-	-	1834.7724
$(x_{16})^{2+}$	917.8819	-	-	917.8802	-
$(x_{17})^{2+}$	982.4032	-	-	-	982.4109
$(x_{18})^{2+}$	1025.9192	-	-	1025.9174	-
$(y_1-NH_3)^+$	130.0863	130.0852	-	-	-
$(y_2)^+$	147.1128	147.1118	-	147.1131	-
$(y_2)^+$	276.1554	276.1535	-	276.1567	276.1552
$(y_2-H_2O)^+$	258.1448	258.1423	-	-	258.1446
$(y_3)^+$	363.1874	363.1841	-	363.191	363.1873
$(y_3-H_2O)^+$	345.1769	345.1736	-	312.1572	345.1761
$(y_4)^+$	477.2304	477.2279	-	477.234	477.2304
$(y_4-H_2O)^+$	459.2198	459.216	-	-	-
$(y_5)^+$	590.3144	590.3102	-	590.3171	590.3146
$(y_5-H_2O)^+$	572.3039	572.2991	-	-	-
$(y_6)^+$	691.3621	691.3597	-	691.3656	691.3628
$(y_6-H_2O)^+$	673.3515	673.3452	-	673.3469	-
$(y_6-NH_3)^+$	674.3355	-	-	674.3389	674.3353
$(y_7)^+$	872.3761	872.3743	-	872.3772	-
$(y_7-H_3PO_4)^+$	774.3992	774.3919	-	-	-
$(y_7-HPO_3)^+$	792.4098	792.4053	-	-	-

$(y_8)^+$	929.3976	929.3898	-	929.398	929.3981
$(y_8\text{-}H_3PO_4)^+$	831.4207	831.4198	-	-	831.4218
$(y_8\text{-}HPO_3)^+$	849.4312	849.4238	-	-	-
$(y_9)^+$	986.419	986.411	986.42	986.4207	986.4199
$(y_9)^{2+}$	493.7132	-	-	-	493.713
$(y_9\text{-}H_3PO_4)^+$	888.4421	888.4356	-	-	888.4432
$(y_9\text{-}HPO_3)^+$	906.4527	906.4468	-	-	-
$(y_{10})^+$	1149.4824	1149.4724	-	1149.4848	1149.4823
$(y_{10}\text{-}H_2O)$	1131.4718	-	-	1131.4611	1131.449
$(y_{10}\text{-}H_3PO_4)^+$	1051.5055	1051.4957	-	-	1051.5084
$(y_{10}\text{-}HPO_3)^+$	1069.516	1069.5068	-	-	-
$(y_{11})^+$	1262.5664	1262.554	-	-	1262.5696
$(y_{11}\text{-}H_3PO_4)^+$	1164.5895	1164.5788	-	-	1164.5892
$(y_{11}\text{-}HPO_3)^+$	1182.6001	1182.5899	-	-	-
$(y_{12})^+$	1365.5756	1365.5626	-	1365.5748	1365.5757
$(y_{12}\text{-}H_2O)^+$	1347.565	-	-	1347.5654	-
$(y_{12}\text{-}H_3PO_4)^+$	1267.5987	1267.5947	-	-	1267.5892
$(y_{12}\text{-}HPO_3)^+$	1285.6093	1285.5977	-	-	-
$(y_{13})^+$	1494.6182	1494.605	1494.62	-	1494.6227
$(y_{13})^{2+}$	747.8127	-	-	-	747.8109
$(y_{13}\text{-}H_3PO_4)^+$	1396.6413	1396.6336	-	-	1396.6473
$(y_{13}\text{-}HPO_3)^+$	1414.6519	1414.6385	-	-	-
$(y_{14})^+$	1581.6502	1581.6359	-	-	1581.6519
$(y_{14})^{2+}$	791.329	-	-	791.3297	791.3296
$(y_{14}\text{-}H_2O)^{2+}$	782.3235	-	-	-	782.3274
$(y_{14}\text{-}H_3PO_4)^+$	1483.6733	1483.6593	-	-	1483.6741
$(y_{14}\text{-}H_3PO_4)^{2+}$	742.3403	742.339	-	-	742.341
$(y_{14}\text{-}HPO_3)^+$	1501.6839	1501.6695	-	-	-
$(y_{15})^+$	1694.7343	1694.7207	-	1694.7318	1694.7355
$(y_{15}\text{-}H_2O)^+$	1676.7237	1676.7178	-	-	-
$(y_{15}\text{-}H_3PO_4)^+$	1596.7574	1596.7414	-	-	1596.7551
$(y_{15}\text{-}H_3PO_4)^{2+}$	798.8823	798.8768	-	-	-
$(y_{16})^+$	1808.7772	1808.7616	1808.77	-	1808.7869
$(y_{16})^{2+}$	904.8925	-	904.89	904.8969	904.8954
$(y_{16}\text{-}H_3PO_4)^+$	1710.8003	1710.7858	-	-	1710.8037
$(y_{16}\text{-}H_3PO_4)^{2+}$	855.9038	855.8991	-	-	-
$(y_{17})^+$	1937.8198	1937.8226	-	-	-
$(y_{17})^{2+}$	969.4135	-	-	969.4134	969.4153
$(y_{17}\text{-}H_3PO_4)^+$	1839.8429	1839.8286	-	-	-
$(y_{17}\text{-}H_3PO_4)^{2+}$	920.4251	920.4184	-	-	-
$(y_{18})^{2+}$	1012.9296	-	-	-	1012.9292
$(y_{18}\text{-}H_3PO_4)^{2+}$	963.9411	963.935	-	-	963.9385
$(z_1)^+$	131.0941	-	-	131.0951	-
$(z_2)^+$	260.1367	-	-	260.1383	-
$(z_2+1)^+$	261.1445	-	261.14	-	-

$(z_3)^+$	347.1667	-	-	347.1701	-
$(z_3+1)^+$	348.1745	-	348.18	-	-
$(z_4)^+$	461.2116	-	461.21	461.2144	461.2116
$(z_5)^+$	574.2957	-	574.3	574.3007	-
$(z_6)^+$	675.3434	-	675.34	675.3469	-
$(z_7)^+$	856.3574	-	856.36	856.3608	-
$(z_8)^+$	913.3788	-	913.38	913.3818	-
$(z_9)^+$	970.4003	-	970.4	-	-
$(z_{10})^+$	1133.4636	-	1133.47	1133.4644	-
$(z_{11})^+$	1246.5477	-	1246.55	1246.5491	-
$(z_{13})^+$	1478.5995	-	1478.6	-	-
$(z_{17})^+$	1921.8011	-	-	1921.8014	-
$(z_{17}+2)^+$	1923.8167	-	1923.81	-	-

Table S6. Modification retention ratios for each fragment containing the modified AA obtained in CID, ETD, EAD and UVPD for the $[M+2H]^{2+}$ MK9pT and DK8pTsY peptides. *[- : not detected]*

**MK9pT**				
	CID	ETD	EAD	UVPD
a_8-1	-	-	1	-
b_7	-	-	1	-
c_7	-	1	1	-
c_8	-	1	-	-
x_3	-	-	1	-
x_4+1	-	-	-	1
x_5	-	-	-	1
x_5+1	-	-	1	-
x_6	-	-	1	-
y_3	0.340	-	0.558	0.461
y_4	0.264	-	0.396	0.234
y_5	0.420	1	0.733	0.460
y_6	0.450	1	0.710	0.511
y_7	0.389	1	1	0.545
y_8	0.436	1	1	0.590
z_3+1	-	1	-	-
z_4	-	-	1	1
z_5	-	1	1	-
z_6	-	1	1	1
z_7	-	1	1	-
z_7+1	-	-	1	-
z_8	-	1	-	-
z_8+1	-	-	-	1
z_9	-	1	-	-

**DK8pTpY**				
	CID	ETD	EAD	UVPD
a_3	-	-	1	1
a_4	-	-	1	-
a_5	-	1	-	-
a_6+1	-	-	1	1
a_7	-	-	1	1
a_7+1	-	1	-	-
b_3	0.316	-	1	0.392
b_4	0.268	-	1	1
b_5	0.504	-	-	1
b_6	-	-	1	-
b_7	-	-	1	0.357
c_3	-	-	1	-
c_3-1	-	-	-	1
c_4	-	-	1	-
c_5	-	-	1	-
c_6	-	1	1	1
c_7	-	1	1	-
x_4	-	-	1	-
x_4+1	-	-	1	-
x_5	-	-	1	1
x_5+1	-	1	-	-
x_6+1	-	-	1	1
x_6-1	-	-	1	-
y_3	1	-	1	1
y_4	1	-	1	1
y_5	0.999	-	1	1
y_5-1	-	1	-	-
y_5-NH_3	-	-	-	1
y_6	0.062	1	1	0.048
y_7	0.868	-	1	0.811
y_7-1	-	-	1	-
z_3	-	1	1	-
z_4	-	1	1	-
z_5	-	1	1	-
z_6	-	1	1	1
z_7	-	-	1	-
z_8	-	1	-	-

Table S7. Modification retention ratios for each fragment containing the modified AA obtained in CID, ETD, EAD and UVPD for the triply protonated $[M+3H]^{3+}$ IK19pS and IK19pT peptides. - : not detected

**IK19pS**				
	CID	ETD	EAD	UVPD
a_6	-	1	1	-
a_7	-	-	1	-
a_7+1	-	-	1	-
a_8	-	-	1	-
a_8+1	-	1	-	-
a_{10}	-	-	1	1
a_{11}	-	-	1	-
a_{13}	-	-	-	0.078
a_{14}	-	-	-	1
a_{15}	-	-	1	1
a_{16}	-	-	1	1
a_{16}+1	-	1	-	-
a_{18}	-	-	1	1
b_6	0	-	-	0
b_7	0	-	1	0
b_8	0.168	-	1	0.394
b_9	0	-	-	-
b_{10}	0.195	-	-	0.357
b_{11}	0	-	-	-
b_{12}	0.312	-	-	-
b_{14}	0.206	-	-	-
b_{15}	0	-	-	0.469
b_{15}^{2+}	-	1	-	-
b_{16}	-	-	1	0.679
b_{16}^{2+}	-	1	-	-
b_{17}	-	-	-	0.542
b_{18}	-	-	-	0.631
c_6	-	-	1	-
c_7	-	-	1	-
c_8	-	-	1	-
c_9	-	1	-	1
c_{10}	-	1	1	-
c_{11}	-	1	1	-
c_{12}	-	1	1	-

**IK19pT**				
	CID	ETD	EAD	UVPD
a_{10}+1	-	1	-	-
a_{13}	-	-	1	0.078
a_{14}	-	-	1	1
a_{15}	-	-	1	0.317
a_{16}	-	-	-	1
a_{17}	-	-	1	
a_{18}	-	-	1	1
b_{14}	0	-	-	0.791
b_{15}	0	-	-	0.140
b_{16}	-	-	-	0.638
b_{17}	-	-	-	0.513
b_{18}	-	-	-	0.701
c_{11}	-	1	-	-
c_{13}	-	1	1	1
c_{14}	-	1	1	-
c_{15}	-	1	1	-
c_{16}	-	1	1	1
c_{17}	-	1	1	1
c_{18}	-	1	1	-
x_7	-	-	-	0
x_8	-	-	1	-
x_{6+1}	-	-	1	1
x_{6-1}	-	-	1	-
x_{10}	-	-	-	1
x_{11}	-	-	-	1
x_{12}	-	-	1	1
x_{13}^{2+}	-	-	1	-
x_{14}	-	-	-	1
x_{14}^{2+}	-	-	1	1
x_{15}	-	-	-	1
x_{15}^{2+}	-	-	1	-
x_{16}	-	-	-	1
x_{16}^{2+}	-	-	1	-
x_{17}^{2+}	-	-	-	1

c_{12}+2	-	-	-	1
c_{13}	-	1	1	1
c_{14}	-	1	1	-
c_{15}	-	1	1	-
c_{16}	-	1	1	1
c_{17}	-	1	1	1
c_{18}	-	1	1	-
x_{14}	-	-	-	1
x_{14} $^{2+}$	-	-	1	-
x_{15}	-	-	1	1
x_{15} $^{2+}$	-	-	1	-
x_{16}	-	-	-	1
x_{16} $^{2+}$	-	-	1	-
x_{17} $^{2+}$	-	-	1	-
y_{14}	0.462	-	1	0.683
y_{14} $^{2+}$	0	-	-	-
y_{15}	0.329	-	1	0.719
y_{15} $^{2+}$	0	-	-	-
y_{15}-NH_3 $^{2+}$	-	-	1	-
y_{16}	0.501	-	1	0.734
y_{16} $^{2+}$	0	1	-	-
y_{16}-NH_3 $^{2+}$	-	-	1	-
y_{17}	0.599	-	-	1
y_{17} $^{2+}$	0	-	-	-
y_{17}-NH_3	-	-	1	-
y_{17}-NH_3 $^{2+}$	-	-	1	-
y_{18} $^{2+}$	0.181	-	1	-
z_7	-	1	-	-
z_{17}+1	-	1	-	-
z_{17}+2 $^{2+}$	-	1	-	-
z_{18} $^{2+}$	-	-	1	-

x_{18} $^{2+}$	-	-	1	-
y_7	0.082	-	1	-
y_8	0.122	-	1	0.781
y_9	0.201	1	1	0.747
y_9 $^{2+}$	-	-	-	1
y_{10}	0.244		1	0.699
y_{10} $^{2+}$	-	-	-	0.848
y_{10}-H_2O	-	-	1	-
y_{11}	0.218	-	-	0.895
y_{12}	0.222	-	1	0.671
y_{12}-H_2O	-	-	1	-
y_{13}	0.214	1	-	0.565
y_{13} $^{2+}$	-	-	-	1
y_{14}	0.456	-	1	0.683
y_{14} $^{2+}$	0	-	1	0.828
y_{15}	0.457	-	1	0.324
y_{15} $^{2+}$	0	-	-	-
y_{16}	0.312	1	-	0.493
y_{16} $^{2+}$	0	1	1	1
y_{17}	0.538	-	-	-
y_{17} $^{2+}$	0	-	1	1
y_{18} $^{2+}$	0	-	-	1
z_7	-	1	1	-
z_8	-	1	1	-
z_9	-	1	-	-
z_{10}	-	1	1	-
z_{11}	-	1	1	-
z_{13}	-	1	-	-
z_{17}	-	-	1	-
z_{17}+2	-	1	-	-
